# A role for glycolipid biosynthesis in severe fever with thrombocytopenia syndrome virus entry

**DOI:** 10.1371/journal.ppat.1006316

**Published:** 2017-04-07

**Authors:** Mary Jane Drake, Benjamin Brennan, Kenneth Briley Jr, Stephen M. Bart, Eric Sherman, Agnieszka M. Szemiel, Madeleine Minutillo, Frederic D. Bushman, Paul Bates

**Affiliations:** 1 Department of Microbiology, Perelman School of Medicine, University of Pennsylvania, Philadelphia, Pennsylvania, United States of America; 2 MRC-University of Glasgow Centre for Virus Research, University of Glasgow, Glasgow, United Kingdom; Harvard Medical School, UNITED STATES

## Abstract

A novel bunyavirus was recently found to cause severe febrile illness with high mortality in agricultural regions of China, Japan, and South Korea. This virus, named severe fever with thrombocytopenia syndrome virus (SFTSV), represents a new group within the *Phlebovirus* genus of the *Bunyaviridae*. Little is known about the viral entry requirements beyond showing dependence on dynamin and endosomal acidification. A haploid forward genetic screen was performed to identify host cell requirements for SFTSV entry. The screen identified dependence on glucosylceramide synthase (*ugcg*), the enzyme responsible for initiating *de novo* glycosphingolipid biosynthesis. Genetic and pharmacological approaches confirmed that UGCG expression and enzymatic activity were required for efficient SFTSV entry. Furthermore, inhibition of UGCG affected a post-internalization stage of SFTSV entry, leading to the accumulation of virus particles in enlarged cytoplasmic structures, suggesting impaired trafficking and/or fusion of viral and host membranes. These findings specify a role for glucosylceramide in SFTSV entry and provide a novel target for antiviral therapies.

## Author summary

As obligate, intracellular parasites, viruses depend upon numerous host-derived factors to replicate. For many bunyaviruses, these pro-viral host factors are largely uncharacterized. In the present study, we used an unbiased genetic screening strategy to identify human genes that facilitate the entry process of a newly discovered pathogenic bunyavirus, severe fever with thrombocytopenia syndrome virus (SFTSV). Our screen and subsequent validation confirmed a role for the glucose-modified lipid, glucosylceramide (GlcCer), in the entry of SFTSV. We found that depletion of GlcCer in the cell leads to the accumulation of virus particles in membranous compartments, suggesting that the virions cannot properly access the cytoplasm to initiate replication. Clinically available compounds that target GlcCer formation may provide a novel antiviral therapeutic strategy for this newly emerged virus.

## Introduction

Emerging viruses pose a public health threat that is increasing with rapid global transit and continued urbanization. Technological advancements have made it easier to identify new viral threats and disseminate information to the public. However, the development of antiviral therapies and vaccines to these new threats remains slow. In 2011, a novel virus was reported causing severe fever and thrombocytopenia in China, with a ~10% mortality rate among hospitalized patients [1–3]. The virus, named severe fever with thrombocytopenia syndrome virus (SFTSV), was found to be a novel bunyavirus belonging to the *Phlebovirus* genus. Other notable members include Rift Valley fever virus (RVFV) and Uukuniemi virus (UUKV) [4,5]. Similar to other phleboviruses, SFTSV is transmitted by biting insects, specifically *Ixodidae* ticks, which were found to carry viruses highly similar (>93%) to those from human isolates [6]. Since 2011, epidemiological studies have found 0.8–3.6% of people are seropositive for SFTSV in endemic regions and seroconversion rates of 45–70% in livestock [7–9] revealing a much wider distribution than previously thought. Further study showed that the geographic distribution of SFTSV extended into South Korea and Japan [10–12]. Since its discovery, SFTSV has come to be better understood clinically [13,14], but many fundamental aspects of the virus biology remain to be elucidated.

The *Bunyaviridae* family of negative-sense RNA viruses is large and diverse with 5 genera and over 300 species identified to date [15]. The tripartite genome is comprised of the S, M, and L segments, which encode the nucleocapsid, glycoproteins, and RNA-dependent RNA polymerase, respectively. In addition to the structural proteins, some bunyaviruses, including SFTSV, also encode non-structural proteins that antagonize host innate immune mechanisms [16–19]. The viral glycoproteins G_N_ and G_C_ form a heterodimer on the surface of virions, and are both necessary and sufficient for viral entry [15].

Studies on the entry of bunyaviruses have identified a requirement for endocytosis and acidification of endosomes for productive virus infection [15]. SFTSV has been shown similarly to require a dynamin-dependent endocytic process and endosomal acidification for efficient entry [20,21]. Additionally, the C-type lectin DC-SIGN has been reported to be a receptor for phleboviruses in dendritic cells (UUKV, RVFV) [22,23], and Raji cells overexpressing DC-SIGN or DC-SIGN-related protein have enhanced susceptibility to SFTSV-glycoprotein mediated infection [20,21]. However, given the limited expression of DC-SIGN and the contrasting wide tropism of SFTSV, additional host factors are likely involved in receptor-mediated endocytosis of SFTSV.

Phylogenetic analysis of the phlebovirus genus has revealed that SFTSV shares only 30–35% glycoprotein amino acid similarity (16–20% identity) with RVFV and UUKV [1,5], and represents a new group within the genus. Based on this difference, SFTSV may have unique requirements for entry into mammalian cells.

In order to identify host factors required for the efficient entry of SFTSV, we performed a forward genetic screen in a human haploid cell line (HAP1) [24–27]. Previous HAP1 screens have been successful at elucidating key requirements of virus entry, including the Ebola virus receptor NPC1 [26] the need for cholesterol in hantavirus internalization and membrane fusion [27,28], and more recently, the identification of the Adeno-Associated Virus receptor AAVR [29]. The present HAP1 screen identified glucosylceramide synthase (*ugcg*, referred to as UGCG) as being essential for the efficient entry of SFTSV. UGCG is a Golgi-resident transmembrane protein that functions as the initiation step of glycosphingolipid (GSL) biosynthesis and is required for the synthesis of gangliosides, globosides, and other cellular GSL species. Using genetic and pharmacological approaches, UGCG expression and enzymatic activity was confirmed to be required for efficient SFTSV entry. Mechanistic studies identified a role for UGCG in the proper trafficking and/or fusion of incoming virions, as cells treated with UGCG inhibitors caused virus particles to accumulate in large cytoplasmic structures. Together, these studies are the first to use an unbiased genetic screen to discover host factors required for SFTSV infection and provide a possible target for therapeutic intervention.

## Results

### Haploid forward genetic screen for SFTSV entry factors identifies candidate genes

**Fig 1:**
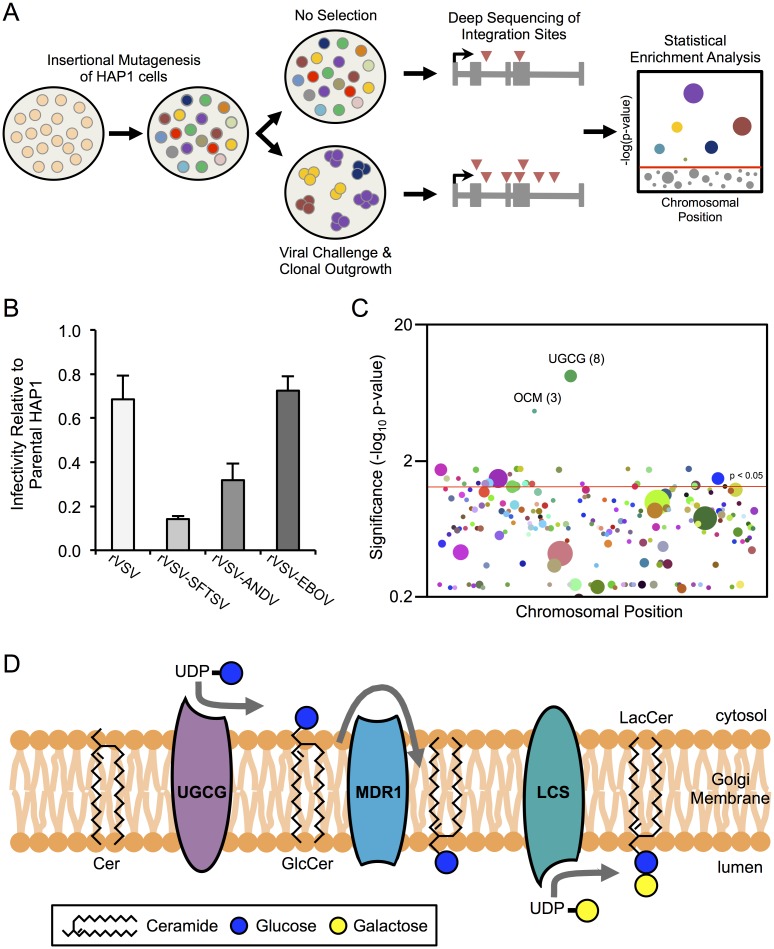
Haploid genetic screen identifies SFTSV entry factors. **(A)** Overview of haploid genetic screen. Details provided in Materials and Methods. **(B)** Parental (non-mutagenized) HAP1 cells and mutagenized HAP1 cells surviving rVSV-SFTSV selection (HAP1-SFTSV^R^) were subject to a single-cycle infection (8–10hr) with a panel of recombinant vesicular stomatitis viruses (rVSVs) encoding various viral glycoproteins. Infection in HAP1-SFTSV^R^ cells is normalized to infection in the parental HAP1 cells. SFTSV = severe fever with thrombocytopenia syndrome virus, ANDV = Andes virus, EBOV = Ebola virus. **(C)** Statistical Enrichment Analysis was carried out using the Chi-Square Exact Test with false discovery rate correction. Each dot represents a unique gene identified from integration site mapping in the HAP1-SFTSV^R^ population. The size of the dot, and number in parenthesis when provided, reflects the number of unique integration sites found within that particular gene. For clarity, only genes with 3 or more unique integration sites are plotted. **(D)** Schematic of glucosylceramide synthesis. GlcCer is synthesized by the membrane-bound UGCG on the cytosolic face of the *cis*-Golgi. The polar head of GlcCer is then flipped to the lumenal side by the transporter MDR1, where it can be further modified by lactosylceramide synthase (LCS) to form lactosylceramide, the core building block of most cellular glycosphingolipids.

To identify host factors that are required for entry of SFTSV, we performed a forward genetic screen in HAP1 cells, a human haploid cell line [26] (Fig 1A). HAP1 cells were mutagenized with a lentiviral gene-trap vector to create a library of genetic knockouts by insertional mutagenesis. Viral challenge of these cells creates a genetic bottleneck, whereby cells harboring mutations in pro-viral genes undergo positive selection. Comparison of mutations in the virus-challenged population to an unselected control population allows for assessment of differences in mutational frequency across the genome. Approximately 75 million mutagenized HAP1 cells were infected with a recombinant vesicular stomatitis virus (VSV) encoding the glycoprotein of SFTSV (strain HB29, rVSV-SFTSV) in place of VSV G. Challenge of non-mutagenized HAP1 cells with rVSV-SFTSV or mutagenized HAP1 cells with wild-type VSV left no surviving cells 7–10 days post infection. In contrast, mutagenized HAP1 cells challenged with rVSV-SFTSV resulted in several hundred surviving colonies, suggesting that survival was due to mutations in genes essential for SFTSV glycoprotein-mediated entry. Following expansion of surviving colonies, cells were collected and either stored at -80°C for additional cellular assays, or genomic DNA was extracted for deep sequencing of the vector integration sites.

To determine whether the cells surviving rVSV-SFTSV infection were indeed refractory to SFTSV-mediated entry, the surviving population and parental HAP1 cells were challenged in parallel with a panel of recombinant VSVs (Fig 1B). Infection of the surviving population with rVSV-SFTSV was greatly reduced compared to parental HAP1 cells, with only 15% residual infection. In contrast, infection with rVSV or an rVSV expressing the glycoprotein of Ebola virus instead of VSV G (rVSV-EBOV) was only modestly reduced in the surviving population compared to the parental cells. Infection with an rVSV expressing the glycoprotein of Andes virus (rVSV-ANDV), a distantly related bunyavirus, showed a intermediate phenotype with 32% residual infection, suggesting potential overlapping requirements for entry of these bunyaviruses. These data indicate that the screen selected pro-viral factors affecting SFTSV entry and not VSV replication.

Using genomic DNA isolated from the rVSV-SFTSV-surviving population, lentiviral integration sites were mapped by deep sequencing of HIV-human genome junctions [30]. The frequency of integration sites within each gene in the surviving population was compared to an unselected control population. A Chi-Square Exact Test with false-discovery rate correction was used to identify genes that were enriched for mutations compared to the control (Fig 1C, S1 Table). The statistical analysis identified glucosylceramide synthase (*UGCG*, UDP-Glucose Ceramide Glucosyltransferase) as a top hit (p-value = 3.7x10^-9^), containing 8 independent integration sites in the set of intragenic integrations sites in the selected cells (N = 4,502).

UGCG catalyzes the first step in glycosphingolipid (GSL) biosynthesis, by attaching a glucose residue to the 1-hydroxyl group of ceramide, forming glucosylceramide (Fig 1D). This forms the backbone for subsequent sugar addition and modification to form the cellular repertoire of gangliosides and additional GSL classes. Given the previously documented roles for GSLs and other glycosylated molecules in the entry of polyomaviruses, rotaviruses, and other viruses [31–33], we focused our subsequent analysis on discerning if UGCG affected SFTSV-glycoprotein mediated entry and the potential role for GSLs in bunyavirus entry as well.

### Genetic manipulation of UGCG validates role for UGCG expression in rVSV-SFTSV entry

**Fig 2:**
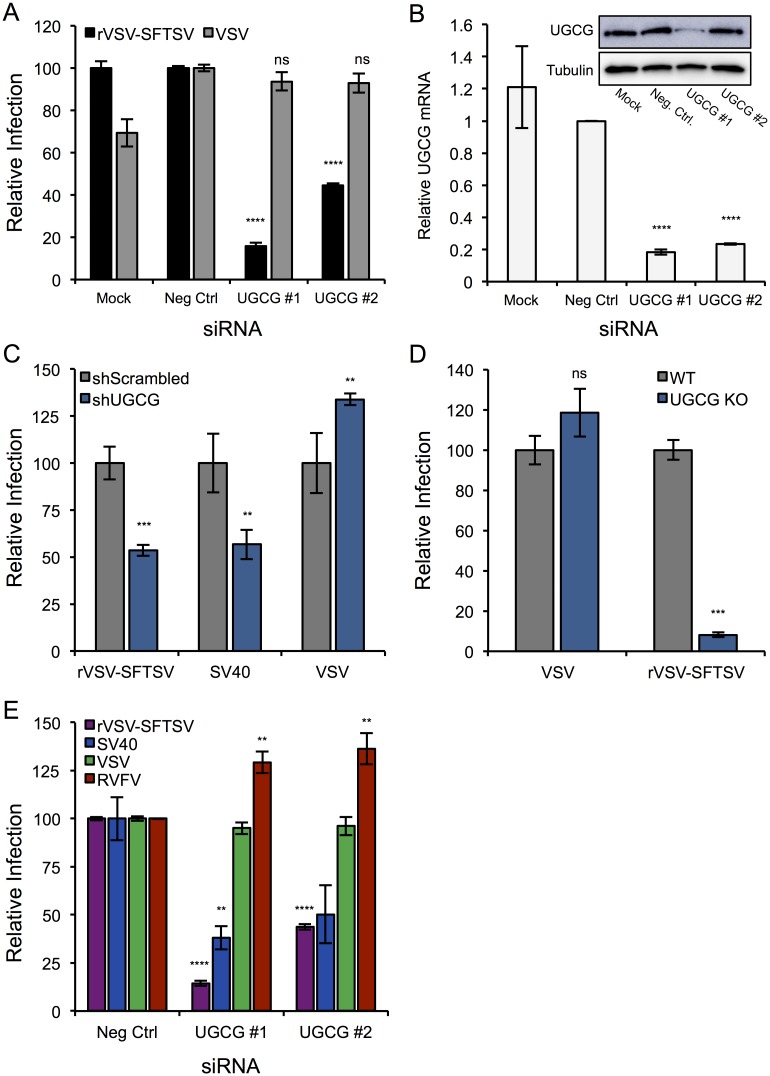
RNAi confirms role for UGCG in SFTSV entry. **(A)** U-2 OS cells were transfected with non-targeting (Neg Ctrl) or UGCG siRNAs and 72 hours post-transfection were infected with rVSV-SFTSV or VSV. Ten hours post-infection cells were harvested, immunostained for VSV M expression, and analyzed by flow cytometry. Infection levels are normalized to the negative control siRNA and expressed as relative infection. Mean ± S.E.M. for 3 independent experiments. **(B)** RNA was collected in parallel at the time of infection from the siRNA treated cells in (A). RT-qPCR was performed and UGCG mRNA was normalized to GAPDH mRNA and expressed relative to the negative control siRNA. Western Blot for UGCG protein expression shown in the inset. **(C)** U-2 OS cells were transduced with lentiviral shRNA constructs containing scrambled or UGCG-targeting shRNAs. Following puromycin selection, cells were infected with rVSV-SFTSV, VSV, or SV40 and harvested 10 hours (rVSV-SFTSV, VSV) or 24 hours (SV40) post-infection, immunostained for viral antigen, and quantified by flow cytometry. Mean ± S.E.M. for 2 independent experiments. **(D)** HAP1 UGCG KO cells or parental HAP1 cells were plated in 24 well plates and the next day infected with rVSV-SFTSV or VSV. Ten hours later, cells were collected, immunostained for VSV M expression, and analyzed by flow cytometry. Infection levels are normalized to parental HAP1 cells and expressed as relative infection. Mean ± S.E.M. for 3 independent experiments. **(E)** U-2 OS cells transfected with negative control or UGCG siRNAs were infected with rVSV-SFTSV, VSV, SV40 or Rift Valley fever virus (RVFV) 72 hours post-transfection. Cells were harvested 10 hours (rVSV-SFTSV, VSV, RVFV) or 24 hours (SV40) post-infection, immunostained for viral antigen, and analyzed by flow cytometry. Mean ± S.E.M. for 3 independent experiments. ** p<0.01, *** p<0.001, **** p<0.0001 using Student’s t-test with Bonferroni correction.

To determine if UGCG plays a role in SFTSV glycoprotein-mediated entry, two unique siRNAs targeting UGCG were tested in parallel with a non-targeting siRNA control (Neg Ctrl). U-2 OS cells were transfected with the siRNAs and 72 hours post-transfection were infected with rVSV-SFTSV or wild-type VSV for a single cycle infection (10hr) (Fig 2A). The siRNAs targeting UGCG both inhibited rVSV-SFTSV infection relative to the non-targeting siRNA, with siUGCG #1 resulting in over 80% reduction in infection (p<0.0001, Student’s t-test). No difference in infection levels was observed for VSV amongst the negative control and UGCG siRNAs, suggesting that phenotype observed with rVSV-SFTSV was due to an entry defect. RT-qPCR for UGCG mRNA and western blotting for UGCG protein expression revealed that both siRNAs were effective at knocking down UGCG mRNA expression, but siUGCG #1 led to a much greater decrease on UGCG protein levels (Fig 2B). The weak effect of siUGCG #2 on UGCG protein expression correlates with the more modest decrease in infection with rVSV-SFTSV (Fig 2A).

To validate the requirement for UGCG using another method, a lentiviral shRNA construct was used to knockdown UGCG expression. Following transduction with the shRNA and 48 hours of puromycin selection to select for the transducing vector, U-2 OS cells were infected with rVSV-SFTSV, VSV, or SV40 (Fig 2C). Entry of the polyomavirus SV40 requires the ganglioside GM1a, which is in a biosynthetic pathway downstream of UGCG, and thus serves as a positive control for knockdown of UGCG activity. The cells receiving the shRNA targeting UGCG (shUGCG) showed a 50% decrease in both rVSV-SFTSV and SV40 infection compared to cells receiving a scrambled shRNA (shScrambled) (rVSV-SFTSV p = 0.0003, SV40 p = 0.0037). This corresponded with an ~50% decrease in UGCG mRNA levels (S1 Fig). VSV infection was slightly increased in shUGCG cells compared to the shScrambled (p = 0.0071).

In addition to RNAi approaches, we also tested the effects of rVSV-SFTSV infection in HAP1 UGCG knockout cells (UGCG KO). Cells were infected with rVSV-SFTSV or VSV for a single replication cycle (Fig 2D). Infection of rVSV-SFTSV was reduced >10-fold in UGCG KO cells compared to parental HAP1 cells (p<0.001), while no significant difference was observed upon VSV infection of both cell types (p = 0.25). These results along with the previous RNAi experiments confirm that UGCG expression is important for the entry of rVSV-SFTSV.

We next tested whether a distantly related phlebovirus, Rift Valley fever virus (RVFV), which has ~20% amino acid identity (~30% similar) to the SFTSV glycoproteins G_N_ and G_C_, was affected by depletion of UGCG using our siRNAs. Similar to previous experiments, treatment with siUGCG #1 or #2 resulted in a decrease in rVSV-SFTSV infection (p<0.0001), and also reduced infection with SV40 as expected from the shRNA experiments (p<0.05) (Fig 2E). In contrast, RVFV infection was slightly enhanced in UGCG-depleted cells (p<0.01), indicating that the requirement for UGCG does not extend to this distantly related phlebovirus.

### UGCG activity is required for efficient rVSV-SFTSV entry

**Fig 3:**
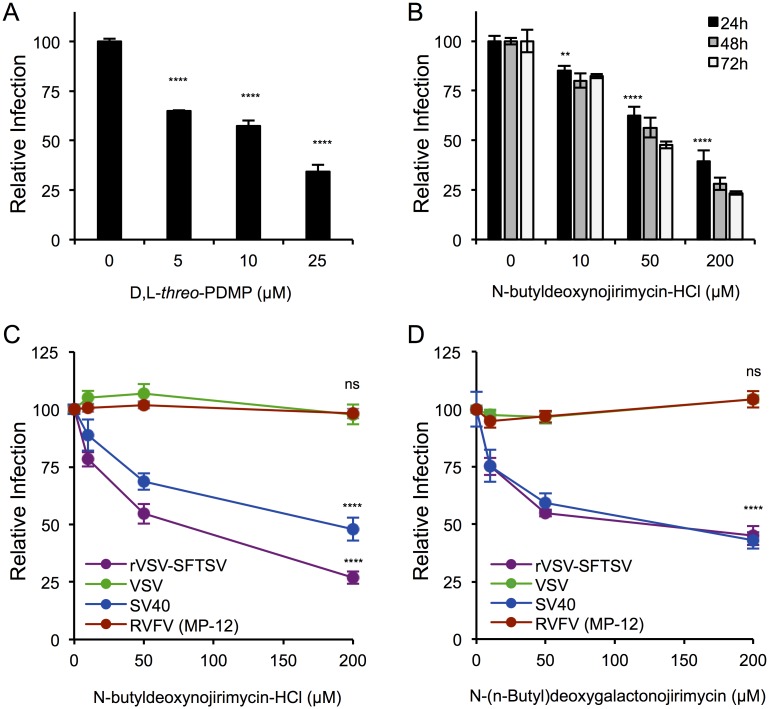
Pharmacological inhibitors of UGCG activity inhibit rVSV-SFTSV entry. **(A)** A549 cells were pre-treated with the UGCG inhibitor D,L-*threo*-PDMP for 24 hours before infection with rVSV-SFTSV. Drug was kept in the media throughout the infection. Ten hours post-infection cells were harvested, immunostained for VSV M, and analyzed by flow cytometry. Mean ± S.E.M. for 3 independent experiments. **(B)** A549 cells were pre-treated with the UGCG inhibitor N-butyldeoxynojirimycin-HCl (NB-DNJ) for 24, 48, or 72 hours before infection with rVSV-SFTSV. Drug was kept in the media throughout the infection. Ten hours post-infection, cells were harvested, immunostained for VSV M, and analyzed by flow cytometry. Mean ± S.E.M. for 3 independent experiments. **(C,D)** A549 cells were pre-treated with NB-DNJ (C) or N-(n-Butyl) deoxygalactonojirimycin (NB-DGJ) (D) for 48 hours before infection with rVSV-SFTSV, VSV, SV40, or RVFV. Drug was kept in the media throughout infection. Ten hours (rVSV-SFTSV, VSV, RVFV) or 24 hours (SV40) post-infection, cells were harvested, immunostained for viral antigen, and analyzed by flow cytometry. Mean ± S.E.M. for 3 independent experiments. **** p<0.0001 using Student’s t-test with Bonferroni correction.

To determine whether the enzymatic function of UGCG was essential for the observed reduction on rVSV-SFTSV infection, several inhibitors of UGCG were tested. A reversible inhibitor, D,L-*threo*-PDMP (PDMP), was added to A549 cells 24 hours prior to infection with rVSV-SFTSV and then maintained in the media throughout the single cycle infection (10hrs) (Fig 3A). A dose dependent decrease in rVSV-SFTSV infection was observed, reaching a 65% reduction at the highest concentration of 25μM (p<0.0001). The second compound tested, N-butyldeoxynojirimycin (NB-DNJ, Miglustat), is FDA approved for the treatment of a lysosomal storage disorder (Type I Gaucher Disease). A549 cells were pretreated for 24, 48, or 72 hours with a range of concentrations of NB-DNJ (10, 50, 200μM) before infection with rVSV-SFTSV in the continued presence of drug (Fig 3B). Across all time points, a dose-dependent decrease in infection with rVSV-SFTSV was observed, with a 60% reduction in infection at the highest dose (200μM) after only 24 hours (p<0.0001). A greater effect was observed after 48 hours (72% reduction) and was maximally reduced by 77% after 72 hours. Of note, the UGCG inhibitors were not cytotoxic under the conditions tested (S2 Fig).

Since the observed effect on rVSV-SFTSV infection could also be due to a defect in replication of the VSV core, cells pretreated with NB-DNJ were infected with wild-type VSV. Also, to ensure that NB-DNJ treatment resulted in decreased ganglioside production, SV40 infection was used as a control. A549 cells were pretreated with NB-DNJ for 48 hours before infection with VSV, SV40, RVFV, or rVSV-SFTSV (Fig 3C). As above, a dose-dependent effect was observed with rVSV-SFTSV (73% decrease at 200μM, p<0.0001), as well as with SV40 (50% decrease at 200μM, p<0.0001). There was no effect seen on VSV infection levels across the range of NB-DNJ concentrations tested (p = 0.69 at 200μM), confirming that the phenotype observed with rVSV-SFTSV was not due to VSV replication. Similar to the siRNA experiments, RVFV was unaffected by NB-DNJ pre-treatment (p = 0.49 at 200μM).

In addition to NB-DNJ, we tested a second imino sugar derivative, N-(n-butyl)deoxygalactonojirimycin (NB-DGJ), that is a chiral isomer of NB-DNJ with enhanced specificity for UGCG. A549 cells were pretreated with NB-DGJ at a range of concentrations (10, 50, or 200μM) for 48 hours before infection with the same viruses as above (Fig 3D). A similar dose-dependent decrease in rVSV-SFTSV and SV40 infection was observed with NB-DGJ treatment (p<0.0001 at 200μM). No effect was seen on VSV and RVFV over the concentrations tested. These data confirm that the effect on rVSV-SFTSV and SV40 infection observed with NB-DNJ was due to targeting of UGCG enzymatic activity. Together these drug studies indicate that the enzymatic activity of UGCG is important for efficient SFTSV glycoprotein-mediated entry and that the changes that occur within the cell to reduce virus entry efficiency occur within 24 hours of drug treatment (Fig 3A and 3B). The requirement for UGCG activity does not appear to extend to RVFV. Additional phleboviruses will need to be tested to fully determine the range of dependency.

### Downstream ganglioside and lactosylceramide biosynthesis does not inhibit rVSV-SFTSV entry

**Fig 4:**
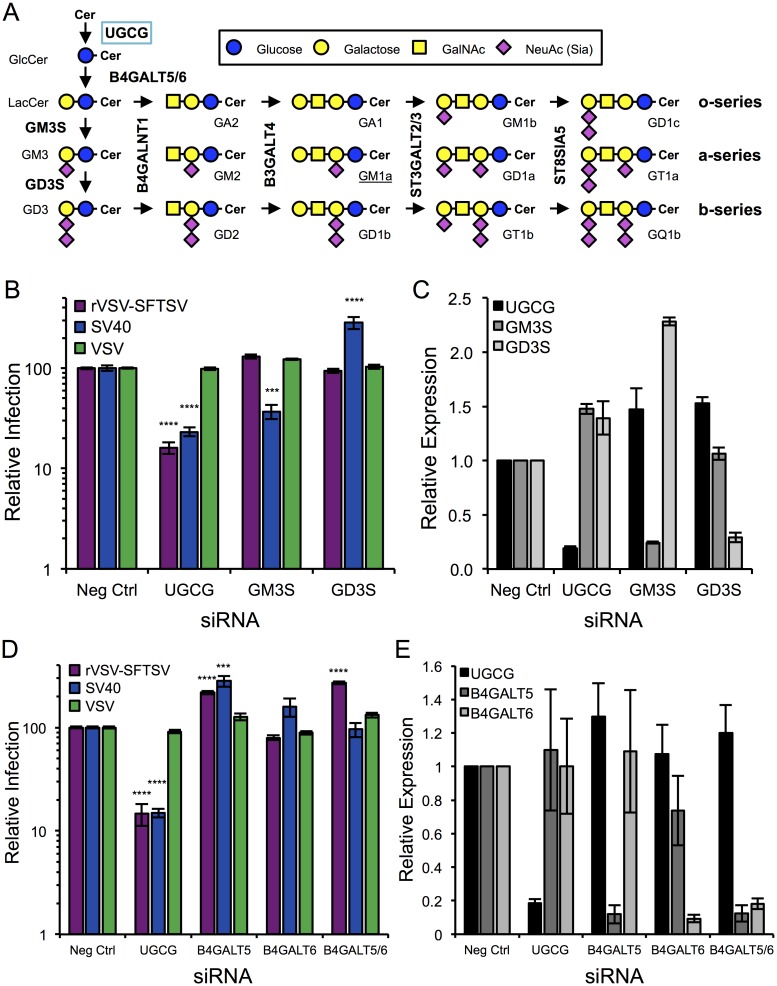
Analysis of downstream glycosphingolipid biosynthesis. **(A)** Diagram of Ganglioside Biosynthesis. Unique enzymes are responsible for the formation of GlcCer, LacCer, GM3 and GD3 (left hand side). These substrates are then modified by common enzymes in a step-wise manner to attach additional sugar moieties. Each of the o-, a-, and b-series branches are a committed pathway, since sialyltransferases cannot convert an a-series ganglioside to a b-series (excluding the conversion of GM3 to GD3). **(B)** U-2 OS cells were transfected with siRNAs targeting UGCG, GM3 Synthase (GM3S), GD3 Synthase (GD3S), or a non-targeting control (Neg Ctrl) and 72 hours later were infected with rVSV-SFTSV, VSV, or SV40. Ten hours (rVSV-SFTSV, VSV) or 24 hours (SV40) post-infection cells were harvested, immunostained for viral antigen, and analyzed by flow cytometry. Mean ± S.E.M. for 3 independent experiments. **(C)** mRNA levels for UGCG, GM3S, and GD3S were determined for (B) using RT-qPCR, normalized to GAPDH mRNA levels, and expressed relative to the negative control siRNA. **(D)** U-2 OS cells were transfected with siRNAs targeting UGCG, B4GALT5, B4GALT6, both B4GALT5 and B4GALT6 (B4GALT5/6), or a non-targeting control (Neg Ctrl). 72 hours post-transfection cells were infected with rVSV-SFTSV, VSV, or SV40. Ten hours (rVSV-SFTSV, VSV) or 24 hours (SV40) post-infection cells were harvested, immunostained for viral antigen, and analyzed by flow cytometry. Mean ± S.E.M. for 3 independent experiments. **(E)** mRNA levels for UGCG, B4GALT5, and B4GALT6 from siRNA-transfected cells were determined by RT-qPCR, normalized to GAPDH mRNA levels, and expressed relative to the negative control siRNA. *** p<0.001, **** p<0.0001 using Student’s t-test with Bonferroni correction.

Glucosylceramide synthesis is the first step in the production of many glycosphingolipid species, including gangliosides (Fig 4A). Various gangliosides can serve as entry receptors for viruses, such as polyomaviruses and rotaviruses [31]. To determine if the observed requirement for UGCG in SFTSV entry was due to the need for a ganglioside receptor, siRNAs targeting key ganglioside biosynthetic enzymes were designed to inhibit various subsets of gangliosides (o-, a-, b-series) (Fig 4B and 4C). Knockdown of GD3 Synthase (GD3S, *ST8SIA1*) in U-2 OS cells had no effect on SFTSV entry and resulted in rVSV-SFTSV infection levels that were similar to the negative control siRNA. In contrast, GD3S siRNA treatment increased SV40 infection (3.7-fold, p<0.001). This increase in SV40 infection in GD3S siRNA treated cells likely results from diversion of GM3 from the b-series ganglioside path into the a-series pathway, which includes the SV40 receptor GM1a. A siRNA targeting GM3 Synthase (GM3S, *ST3GAL5*) was also tested, which results in decreased a- and b-series gangliosides, including GM1a. Following GM3S knockdown in U-2 OS cells, infection with SV40 was decreased 4.5-fold (p<0.0001), while rVSV-SFTSV infections were again similar to the negative control siRNA. For both siRNAs, VSV infection levels also remained similar to the negative control siRNA, indicating there was no effect on VSV entry or replication. As a positive control, siRNAs targeting UGCG were also included and infection of rVSV-SFTSV and SV40 decreased similar to previous experiments. Together, this evidence suggests that complex ganglioside formation is not required for rVSV-SFTSV infection.

Immediately following glucosylceramide formation, GlcCer is flipped from the cytosolic surface of the Golgi complex to the lumenal surface where a galactose moiety is added by lactosylceramide synthase (LCS) to form lactosylceramide (LacCer) (Fig 1D). To test whether the formation of the simple glycolipid LacCer was required for SFTSV entry, siRNAs targeting LCS were obtained (Fig 4D and 4E). Two cellular LCSs exist, B4GALT5 and B4GALT6, with B4GALT5 being more ubiquitous. Knockdown of B4GALT5 lead to a 2-fold increase in rVSV-SFTSV infection (p<0.0001), while knockdown of B4GALT6 had no effect. In case B4GALT5 can compensate for B4GALT6 within the cell due to redundant functions, dual knockdown B4GALT5 and B4GALT6 was performed. Dual knockdown lead to a 2.7-fold increase in rVSV-SFTSV infection (p<0.0001). The observed increases on rVSV-SFTSV infection could potentially be caused by increased GlcCer accumulation, although more experiments will be needed to address the mechanism for the observed phenotype. Surprisingly, an increase on SV40 infection was observed with knockdown of B4GALT5 and B4GALT6, but no effect was observed when both LCSs were targeted. Surface expression of GM1a in siB4GALT5 and siB4GALT6-treated cells was increased compared to negative control siRNA and siB4GALT5/6 treated cells (S3 Fig), potentially explaining the observed increased in infection with SV40.

### UGCG expression and activity are also required for wild-type SFTSV infection

**Fig 5:**
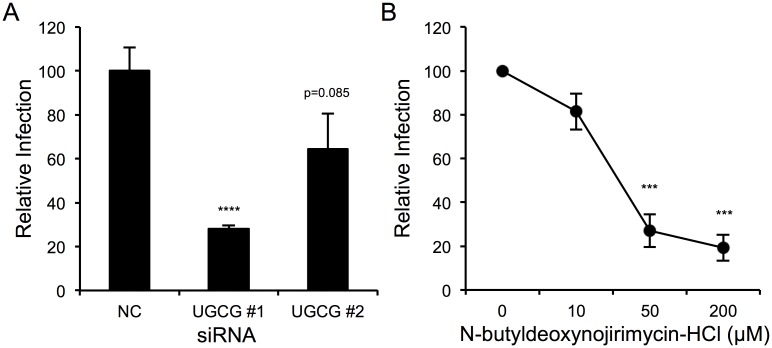
UGCG activity and expression important for wild-type SFTSV infection. **(A)** U-2 OS cells were transfected with siRNAs to UGCG or a non-targeting control and 72 hours later were infected with SFTSV (strain HB29, MOI 0.1). Supernatants from infected cells were collected 48 hours post-infection and viral output was determined by plaque assay. Infectious output is expressed relative to the negative control siRNA. **(B)** U-2 OS cells were pre-treated with NB-DNJ for 48 hours before infection with SFTSV (MOI 5). Supernatants from infected cells were collected 12 hours post-infection and viral output was determined by plaque assay. Infectious output is expressed relative to the untreated control. *** p<0.001, **** p<0.0001 using Student’s t-test with Bonferroni correction.

Since the screen and subsequent validation was performed using a recombinant virus, it was important to determine if UGCG expression and activity were required by a clinical isolate of SFTSV. U-2 OS cells were transfected with siRNAs targeting UGCG and infected 72 hours later with SFTSV (strain HB29, MOI 0.1) (Fig 5A). The infection was allowed to proceed for 48 hours before supernatants were collected and viral titers were determined by plaque assay. Similar to what was observed with rVSV-SFTSV (Fig 2A), wild-type SFTSV showed a 75% decrease in infection with siUGCG #1 compared to the negative control siRNA (p<0.0001). A less robust decrease was observed with siUGCG #2 (35% decrease, p = 0.085), again similar to what was observed with rVSV-SFTSV, likely due to incomplete knockdown of UGCG protein expression.

To test whether UGCG enzymatic activity was required for efficient wild-type SFTSV infection, U-2 OS cells were pretreated with NB-DNJ for 48 hours, and then infected with SFTSV (strain HB29, MOI 5) (Fig 5B). Following 12-hours of infection, supernatants were collected and viral titers determined by plaque assay. A dose-dependent decrease in viral output was observed, with titers being reduced by 80% at the highest dose (200μM, p<0.001). Together, these observations suggest that UGCG expression and activity are required for efficient replication of wild-type SFTSV. Furthermore, the use of rVSV-SFTSV as model for studying SFTSV entry faithfully recapitulates what is seen with the wild-type virus.

### Heartland virus also requires UGCG for efficient entry

**Fig 6:**
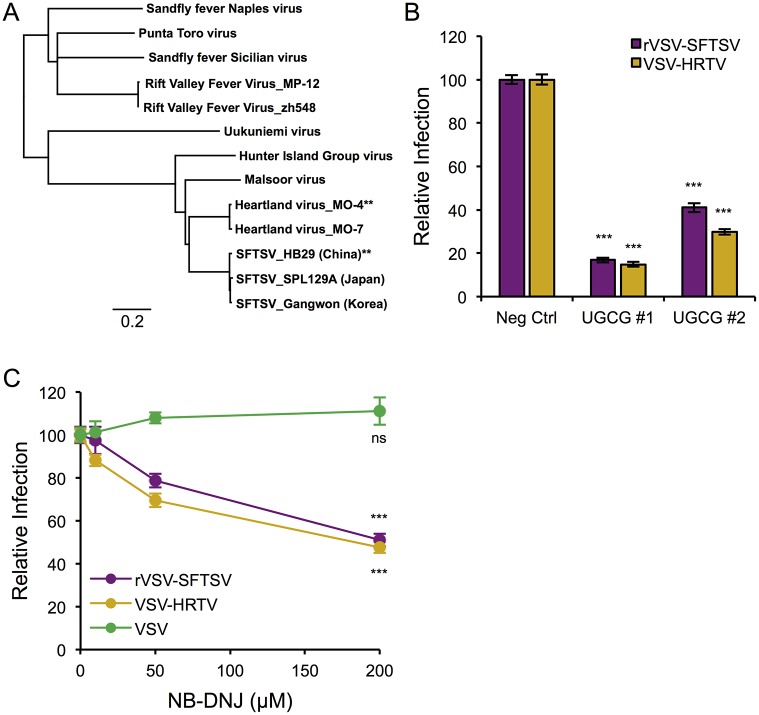
Heartland virus, close relative of SFTSV, also requires UGCG for efficient entry. **(A)** Phylogenetic tree based on the amino acid alignments of various phlebovirus glycoproteins (polyprotein precursor). Scale bar represents genetic distance as a percent difference (0.2 = 20%). Asterisks denote virus sequences used to generate codon-optimized M segments (G_N_-G_C_) for the present study. **(B)** U-2 OS cells were transfected with siRNAs to UGCG or a non-targeting control, then 72 hours later were infected with rVSV-SFTSV or the VSV pseudotype bearing the Heartland virus glycoprotein (VSV-HRTV). Twelve hours post-infection cells were harvested, immunostained for VSV M, and analyzed by flow cytometry. Percent infection was normalized to samples receiving the negative control siRNA and expressed as relative infection. Mean ± S.E.M. for 3 independent experiments. **(C)** U-2 OS cells were pre-treated with NB-DNJ for 48 hours before infection with rVSV-SFTSV, VSV-HRTV or VSV. Drug was kept in the media throughout the infection. Twelve hours post-infection cells were harvested, immunostained for VSV M, and analyzed by flow cytometry. Percent infection was normalized to the untreated control for each virus and expressed as relative infection. Mean ± S.E.M. for 3 independent experiments. *** p<0.001 using Student’s t-test with Bonferroni correction.

Recently, another tick-borne phlebovirus closely related to SFTSV, Heartland Virus (HRTV), was identified as the cause of severe febrile disease in several patients in the Midwestern United States [34]. HRTV is approximately 80% similar (~65% identical) to SFTSV at the amino acid level, and groups with SFTSV on a phylogenetic tree of phlebovirus glycoprotein amino acid alignments (Fig 6A, S4 Fig). A codon-optimized version of the Heartland Virus glycoprotein (strain MO-4) was synthesized and used to generate pseudotypes on a VSV core (VSV-HRTV), similar in structure to our rVSV-SFTSV replication-competent virus. U-2 OS cells were treated with siRNAs targeting UGCG and infected with VSV-HRTV or rVSV-SFTSV (Fig 6B). Infection with VSV-HRTV was reduced by 85% in cells treated with siUGCG #1 (p<0.001), similar to the 83% decrease observed on rVSV-SFTSV infection. Again, infection of cells treated with siUGCG #2 exhibited a less robust decrease, but did inhibit VSV-HRTV by 70% (p<0.001) and rVSV-SFTSV by 59% (p<0.001).

UGCG activity was inhibited using NB-DNJ similar to previous experiments. Following 48 hours of pre-treatment, U-2 OS cells were infected with VSV-HRTV or rVSV-SFTSV with drug maintained throughout the infection (Fig 6C). Similar to previous results, rVSV-SFTSV exhibited a dose-dependent decrease in infection, and consistent with the siRNA experiment, VSV-HRTV infection was negatively impacted by UGCG inhibition with NB-DNJ. At the highest dose of 200μM, VSV-HRTV infection levels were reduced by 53% (p<0.001), on par with 50% reduction observed with rVSV-SFTSV (0<0.001). Together, these results confirm a similar role for UGCG expression and activity on the efficient entry of Heartland virus, a phlebovirus closely related to SFTSV.

### UGCG inhibition does not affect the binding or internalization of rVSV-SFTSV

Virus entry is described by discrete stages, beginning with binding of the virus to a target cell via an attachment factor or receptor. In the case of bunyaviruses, the virion must then be internalized by endocytosis and traffic through the endo-lysosomal network to a low pH compartment, wherein the glycoprotein undergoes a conformation change allowing for fusion of the viral and host membranes [15,35]. In order to determine the stage of the SFTSV entry process where UGCG is required, a qPCR-based binding and internalization assay was employed. The assay can assess relative levels of virus bound to and internalized by normal cells compared to those treated with UGCG-targeting siRNAs or pharmacological inhibitors of UGCG. Cells were chilled to 4°C on ice for 30 minutes before rVSV-SFTSV was added and allowed to bind to cells for 1 hour at 4°C. Following the first hour, a subset of cells was moved to 37°C to allow internalization of bound virions while the remaining cells were left at 4°C. At the conclusion of the second hour, cells that were left at 4°C were washed thoroughly with PBS and either directly lysed to determine the amount of bound virus, or treated with trypsin, washed, and then lysed to remove surface-bound virions in order to define the amount of background in the assay. The cells that had been warmed to 37°C were also trypsinized, washed and then lysed, providing a measure of the amount of internalized virus. Primers that anneal to the negative-strand viral genome were used for reverse-transcription and quantitative PCR (S2 Table).

**Fig 7:**
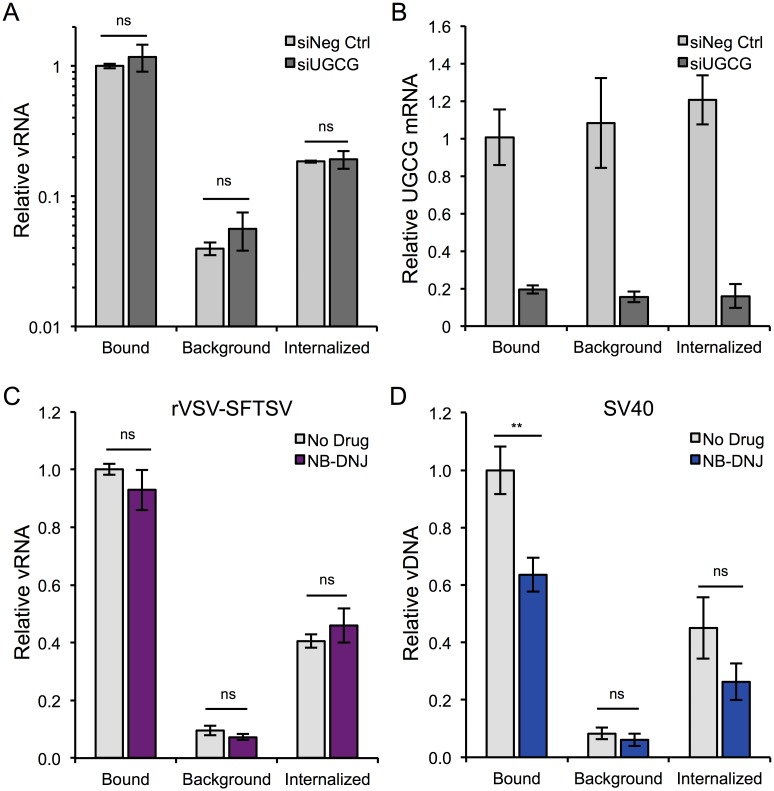
Mechanistic studies on UGCG’s role in SFTSV entry. **(A)** Binding and Internalization Assay. U-2 OS cells were transfected with negative control or UGCG siRNAs, replated the following day into 24 wells dishes, and the assay was performed 72 hours post-transfection. Assay details provided in Materials and Methods. vRNA levels were normalized to GAPDH mRNA levels, and are expressed relative to bound vRNA for the negative control siRNA. Mean ± S.E.M. for 3 independent experiments. **(B)** RNA collected from (A) was also analyzed for UGCG mRNA expression. UGCG mRNA levels were measured by RT-qPCR, normalized to GAPDH mRNA levels, and expressed relative to the negative control siRNA (bound). **(C,D)** The binding and internalization assay was carried out essentially as in (A) with the exception that U-2 OS cells were instead pre-treated with NB-DNJ for 48 hours prior to binding with rVSV-SFTSV (C) or SV40 (D) and qPCR for SV40 genomes did not require reverse transcription. Mean ± S.E.M. for 3 independent experiments. ** p<0.01 using Student’s t-test.

U-2 OS cells were transfected with siRNAs either targeting UGCG (siUGCG #1) or a non-targeting control (siNeg Ctrl), and 3 days later were used to assess binding and internalization levels of rVSV-SFTSV (Fig 7A). Despite efficient knockdown of UGCG in siRNA-transfected cells as measured by qPCR (Fig 7B), there was no observable difference in the efficiency of binding or internalization of rVSV-SFTSV between siRNA treatments. To ensure that this stage of viral entry was indeed unaffected by UGCG inhibition, the assay was repeated with the UGCG inhibitor NB-DNJ. U-2 OS cells were pretreated with NB-DNJ or left untreated for 48 hours before being used to assess binding and internalization levels for rVSV-SFTSV (Fig 7C). Similar to the siRNA-treated cells, there was no observable difference in the relative amount of bound or internalized rVSV-SFTSV in untreated or NB-DNJ-treated cells, suggesting that these early steps in SFTSV entry are not impacted by UGCG inhibition. To verify that the qPCR assay was indeed robust and the observed results with rVSV-SFTSV were accurate, SV40 binding and internalization was measured (Fig 7D). Since NB-DNJ treatment results in decreased synthesis of GM1a and SV40 requires GM1a as its receptor, SV40 should exhibit reduced binding in NB-DNJ-treated cells compared to untreated cells. Consistent with this hypothesis, SV40 binding was significantly reduced by 36% in NB-DNJ treated cells compared to the untreated control (p<0.01). As anticipated because of the reduced binding, internalization of SV40 was also decreased by 42% with NB-DNJ treatment though the observed difference was not statistically significant (p = 0.17). Together, these data demonstrate that UGCG does not function in the very early steps of virus entry for rVSV-SFTSV, including binding and internalization and suggest a role in trafficking or penetration of incoming virions.

### Inhibition of UGCG activity or expression causes virus accumulation at late stages of entry

Previous studies on the entry of SFTSV [20,21] have found that, similar to other bunyaviruses [35], SFTSV requires endocytosis via a dynamin-dependent mechanism and endosomal acidification for entry. Investigations by Lozach *et al* using the distantly related phlebovirus, Uukuniemi virus (~20% glycoprotein a.a. identity), have observed trafficking of incoming virions through Rab5, Rab7 and LAMP1-positive endosomes, respectively [36]. However, no detailed studies on the trafficking of incoming SFTSV virions have been reported.

In order to determine if inhibition of UGCG altered the trafficking or localization of incoming virions, immunofluorescence microscopy was used to observe the distribution of internalized virus particles within the cell. Briefly, A549 cells plated on glass coverslips were treated with NB-DNJ (200μM) for 48 hours or left untreated. After 48 hours, cells were chilled for 30 minutes on ice at 4°C to inhibit endocytosis before addition of rVSV-SFTSV in chilled media and binding of virions by centrifugation at 4°C. Following centrifugation, media was replaced with pre-warmed media (37°C) and placed in a 37°C incubator for the indicated length of time before fixation and immunostaining for viral antigen and various cellular markers.

**Fig 8:**
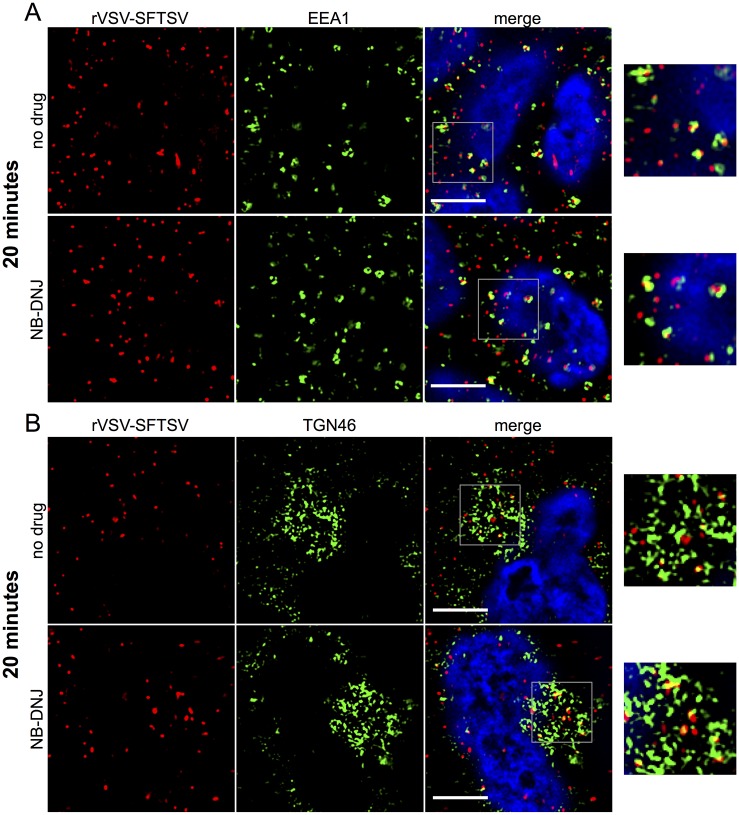
Immunofluorescence microscopy of incoming virus particles, 20 minutes. (**A, B**) A549 cells were plated onto glass coverslips and the following day replaced with media containing NB-DNJ (200μM) or left untreated. Forty-eight hours later, cells were chilled to 4°C on ice, then rVSV-SFTSV was bound by centrifugation (1200xg, 30’, 4°C). Following centrifugation, media was replaced with pre-warmed media (37°C) and the cells placed in a 37°C incubator for 20 minutes before fixation in 2% paraformaldehyde for 10 minutes. Cells were then immunostained for viral antigen (anti-VSV M, red), cellular markers (green), and nuclei stained with DAPI (blue). Images are representative from at least 3 independent experiments. (**A**) A549 cells co-stained for rVSV-SFTSV (red) and early endosome marker EEA1 (green). (**B**) A549 cells co-stained for rVSV-SFTSV (red) and *trans*-Golgi marker TGN46 (green). Boxes indicate zoomed-in regions. Scale bar represents 5μm.

At twenty minutes post-warming, rVSV-SFTSV could be visualized in EEA1+ early endosomes (Fig 8A). Virus was observed to populate EEA1+ early endosomes similarly between untreated and NB-DNJ treated cells. No appreciable overlap was seen between rVSV-SFTSV and Rab7 or LAMP1 at 20 minutes in either untreated or NB-DNJ treated cells (S5 Fig). Virus particles were observed co-localizing with the *trans*-Golgi complex marker TGN46 at 20 minutes under both treatment conditions (Fig 8B).

**Fig 9:**
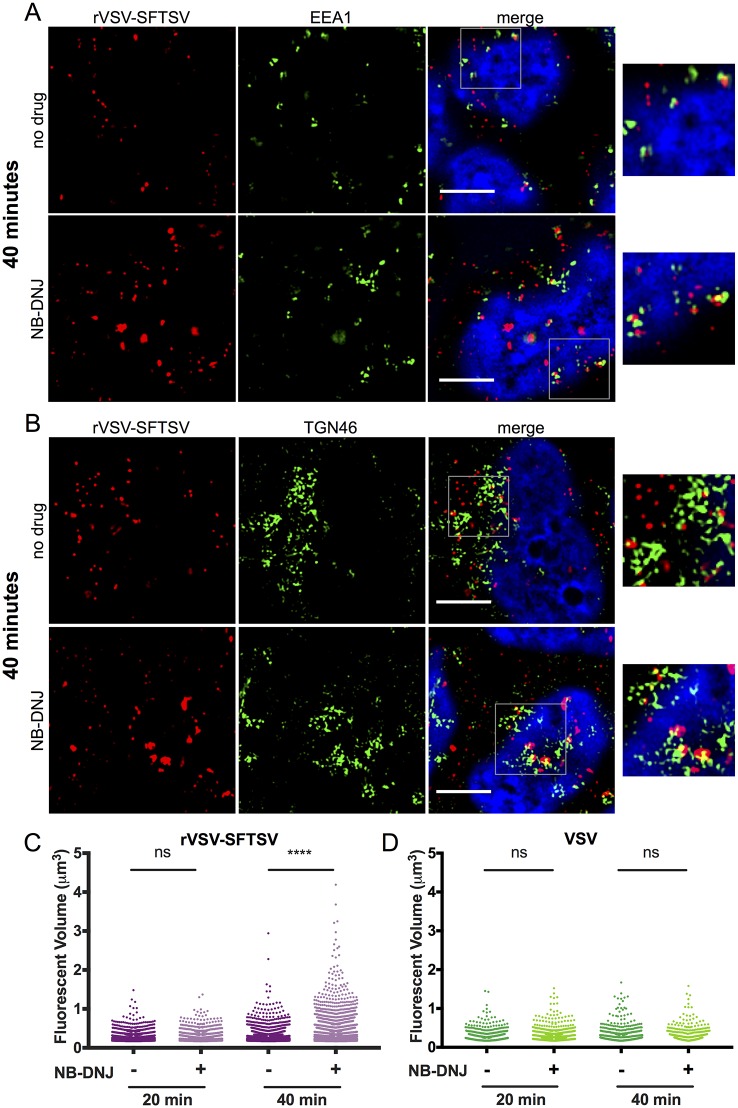
Immunofluorescence microscopy and quantification of incoming virus particles. (**A, B**) A549 cells prepared as described in Fig 8 were fixed 40 minutes post-warming and co-stained for rVSV-SFTSV (red) and the early endosome marker EEA1 (green) (A) or TGN46 (B). Cells were treated with NB-DNJ (bottom panels) or left untreated (top panels). Boxes indicate zoomed-in regions. Scale bar represents 5μm. (**C, D**) Quantitative image analysis was performed to measure the volume of discrete VSV M-stained puncta within z-stack images in untreated and NB-DNJ treated cells at both 20 and 40 minutes post-warming. Puncta were counted for at least 6 independent z-stacks per sample for both rVSV-SFTSV (C) and VSV (D) infected cells. (**** p<0.0001 using Welch’s one-tailed t-test).

At 40 minutes post-warming in untreated A549 cells, rVSV-SFTSV was still observed in EEA1+ endosomes. (Fig 9A, **top panels**). Virus also appeared to localize with TGN46+ compartments (Fig 9B, **top panels**). Extensive co-localization was not seen between rVSV-SFTSV and Rab7 or LAMP1 at 40 minutes post-warming, similar to the 20-minute time point (S6 Fig).

In contrast to untreated cells, at 40 minutes post-warming in NB-DNJ treated cells, rVSV-SFTSV was observed to accumulate in cytoplasmic structures within the cell (Fig 9A and 9B, **bottom panels**). The viral aggregates appeared to have markers of both EEA1 (Fig 9A, bottom panels) and TGN46 (Fig 9B, bottom panels), although not exclusively. The virus containing compartment(s) can be seen with and without EEA1 and TGN46, and the cellular markers do not appear to completely envelop the compartment, suggesting a more complex composition. Due to antibody cross-reactivity, co-staining for both EEA1 and TGN46 within the same infected cell was not possible. Similarly, viral accumulation was also observed at 40 minutes post-warming in A549 cells pre-treated with the highly specific UGCG inhibitor NB-DGJ (S7 Fig).

In order to quantify accumulation of incoming virions, the volume of rVSV-SFTSV-positive puncta was measured by quantitative image analysis. Intensity and volume cutoffs were determined based on immunostaining for viral antigen in uninfected cells and set to exclude >99% of background staining. At 20 minutes post-warming, no significant difference in rVSV-SFTSV virions volumes between untreated and NB-DNJ treated cells was observed (Fig 9C). However, at 40 minutes post-warming the virion puncta volumes in NB-DNJ treated cells were significantly greater than those in untreated cells (p<0.0001, one-tailed Welch’s t-test). In order to ensure the observed phenotype was specific to the SFTSV glycoprotein, virion puncta volumes were also calculated for wild-type VSV-infected cells with or without NB-DNJ pre-treatment. As expected based on the previous infection data, VSV virion puncta volumes were not significantly different between untreated and NB-DNJ treated cells at 20 minutes or 40 minutes post-warming (Fig 9D, S8 Fig).

**Fig 10:**
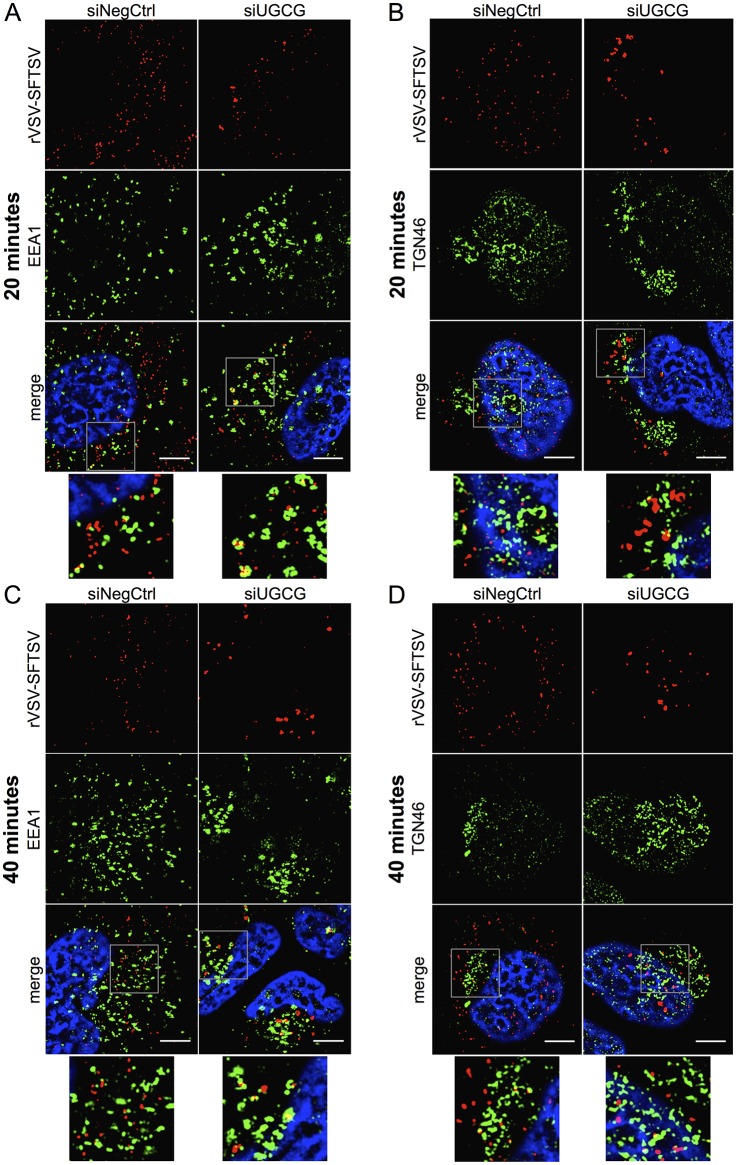
Immunofluorescence microscopy of rVSV-SFTSV particles following UGCG knockdown. **(A-D)** U-2 OS cells were transfected with siRNAs targeting UGCG (siUGCG) or a non-targeting control (siNegCtrl) and plated onto glass coverslips. At 72 hours post-transfection cells were chilled to 4°C on ice and rVSV-SFTSV was bound by centrifugation (1200xg, 30’, 4°C). Following centrifugation, media was replaced with pre-warmed media (37°C) and the cells placed in a 37°C incubator for 20 or 40 minutes before fixation in 1% paraformaldehyde for 15 minutes. Cells were then immunostained for viral antigen (anti-VSV M, red), cellular markers (green), and nuclei stained with DAPI (blue). Images are representative from at least 3 independent experiments. (**A,B**) U-2 OS cells fixed after 20 minutes were co-stained for rVSV-SFTSV (red) and EEA1 (A) or TGN46 (B) (green). (**C,D**) U-2 OS cells fixed after 40 minutes and stained as above. Boxes indicate zoomed-in regions. Scale bar represents 5μm.

To assess whether genetic manipulation of UGCG expression displayed a similar phenotype, rVSV-SFTSV particles were followed upon entry into U-2 OS cells treated with siRNAs targeting UGCG (siUGCG) or a non-targeting control (siNeg Ctrl) (Fig 10, S9 and S10 Figs).

Similar to A549 cells, rVSV-SFTSV virions were observed co-localizing with EEA1+ and TGN46+ compartments (Fig 10). Virions could also be seen in Rab5+ and golgin97+ compartments, additional markers of early endosomes and the *trans*-Golgi network, respectively (S9 Fig). In contrast with A549 cells, rVSV-SFTSV virions entering U-2 OS cells did show some overlap with Rab7+ endosomes at 20 and 40 minutes post-warming (S10 Fig).

Consistent with NB-DNJ treatment, siRNA depletion of UGCG led to rVSV-SFTSV particle accumulation within cells. The aggregates observed under siUGCG conditions also exhibited incomplete co-staining with early endosome and *trans*-Golgi markers, potentially indicating that virus aggregates are enveloped by heterogeneous membranes. Additionally, aggregation of virions could be seen as early as 20 minutes post-warming (Fig 10), whereas a build-up of virions was not observed until 40 minutes post warming in NB-DNJ-treated cells.

Using both pharmacological and genetic approaches, these data suggest that UGCG is important for the proper intracellular trafficking of rVSV-SFTSV virions. While no gross alteration in the localization of virions was observed between treatment and control conditions, inhibition of UGCG led to the accumulation of rVSV-SFTSV virions within the cytoplasm. Whether this represents virus that is halted in a normal compartment for infection or is an aberrant sidetrack remains to be determined.

## Discussion

Genetic screens in haploid human cells are a powerful approach for elucidating virus-host interactions, including identifying critical cellular factors in the virus entry process. Several HAP1 screens have used replication-competent, recombinant vesicular stomatitis viruses (rVSV) encoding orthogonal glycoproteins to discover cellular factors required for the entry of highly pathogenic viruses, including Ebola [26], Andes [27,28], and Lassa viruses [37,38]. The present study utilized an rVSV encoding the glycoprotein of the newly emerged bunyavirus, SFTS virus (rVSV-SFTSV). Challenge of the HAP1 library with rVSV-SFTSV led to a significant enrichment in cells harboring mutations in glucosylceramide synthase (UGCG), a critical component of cellular glycosphingolipid (GSL) biosynthesis. GSLs have been implicated as receptors or attachment factors for a number of different viruses, including the polyomaviruses SV40 (GM1a) and BK virus (GD1b, GT1b), human rotavirus (GM1, histo-blood group antigens [HBGA]), and human norovirus (HBGA) [32,39–43]. We showed that UGCG expression and enzymatic activity are important for SFTSV entry, using both rVSV-SFTSV and wild-type SFTSV infection. Additionally, using pseudotypes bearing the glycoproteins of the closely related Heartland virus, we showed that entry of this emerging virus endemic to the United States was also sensitive to UGCG perturbation.

Given the role of UGCG in GSL biosynthesis, we sought to determine if, similar to the non-envelope viruses previously mentioned, SFTSV also required complex ganglioside formation for entry. siRNAs targeting key ganglioside synthesis enzymes did not affect SFTSV entry. Furthermore, pre-incubating virus with a mixture of complex gangliosides (predominantly GM1a, GD1a, GD1b, GT1b) before infection of U2OS cells did not neutralize rVSV-SFTSV infection, although it did neutralize SV40 in a dose-dependent manner (S11 Fig). These observations confirm that complex ganglioside formation is not necessary for efficient SFTSV entry. However, a number of other GSL species containing different glycosylated cores, such as the globo-series and lacto-series GSLs, were not tested in the present study.

Following GlcCer synthesis by UGCG on the cytosolic face of the *cis*-Golgi, GlcCer is flipped to the lumenal membrane by MDR1 where lactosylceramide synthase (B4GALT5, B4GALT6) appends a galactose moiety to the terminal glucose (Fig 1D) [44–46]. Inhibition of lactosylceramide synthase leads to the accumulation of GlcCer [47,48], and we observed an increase in SFTSV infection when B4GALT5 or B4GALT5 in conjunction with B4GALT6 were down regulated using siRNAs (Fig 4D). Surprisingly, infection with SV40 was also increased when B4GALT5 or B4GALT6 was inhibited, even though GM1a synthesis is downstream of LacCer synthesis. Staining cells treated with siB4GALT5 or siB4GALT6 with fluorescently-tagged cholera toxin subunit B, which also binds to GM1a, was increased compared to cells treated with a non-targeting siRNA or siRNAs to both lactosylceramide synthases (S3 Fig). This could potentially explain the increase in SV40 infection, such that within the time scale of the siRNA knockdown (72 hours) GSL recycling may compensate for inhibition of *de novo* GSL synthesis. More investigations are needed to fully determine the role of GlcCer accumulation and LacCer down-regulation on SFTSV infection, and the role that B4GALT5/6 inhibition plays on the cellular homeostasis of GSL formation.

The entry process for enveloped viruses can be described by discrete stages, beginning with the binding or attachment of virus particles to the host cell surface. For viruses requiring endocytosis, binding to an attachment factor or receptor leads to internalization of the virus particle. The virus will then traffic within the endo-lysosomal network to reach the proper environment (e.g. providing proper low pH, protease cleavage, intracellular receptor, or lipid composition) where the viral and host membrane fusion is triggered, releasing the genomic contents into the cytoplasm for replication. In order to determine if UGCG played a role in the binding or internalization of SFTSV, we performed a qPCR-based binding and internalization assay using both cells treated with siRNAs targeting UGCG or a pharmacological inhibitor of UGCG (NB-DNJ). Under both conditions, neither the binding nor the internalization of rVSV-SFTSV virions was affected by UGCG inhibition. This suggests that UGCG inhibition impedes a post-internalization stage of the entry process. Potential stages include the trafficking of internalized virions to a particular location, priming or maturation of the viral envelope, and/or the fusion of virus-host membranes.

The endocytic trafficking requirements for several bunyaviruses have been investigated, but this study is the first report examining SFTSV glycoprotein-mediated entry. Studies with the phlebovirus Uukuniemi virus (UUKV) by Lozach *et al* sequentially observed UUKV particles in Rab5a, Rab7a, and LAMP1-positive compartments, suggesting UUKV virus penetrates into the host cytoplasm from a late endosomal compartment [36]. In contrast, the orthobunyavirus LaCrosse virus (LACV) and the nairovirus Crimean-Congo hemorrhagic fever virus (CCHFV) were found to only require trafficking to early endosomes and multi-vesicular bodies, respectively [49–51]. In all cases, trafficking into early endosomes was required. Similarly, in the present study we observed rVSV-SFTSV virions co-localizing with the early endosome marker EEA1 at 20 minutes post-warming (Fig 8A). Minimal co-localization of virions with Rab7 or LAMP1 was observed at 20 and 40 minutes post-warming (S5 and S6 Figs) in A549 cells, but some overlap was observed with Rab7 in U-2 OS cells (S10 Fig). While conflicting, these findings suggest that SFTSV may not require trafficking to Rab7 and LAMP1 compartments in certain cell types. In the case of UUKV, despite being observed in Rab7a-positive compartments, expression of the dominant negative Rab7a T22N mutant had no effect on UUKV infection, nor did it inhibit LACV or CCHFV infection [36,49,50]. Further investigation is needed to clarify the requirement of Rab7 on SFTSV entry.

We observed co-localization of rVSV-SFTSV virions with the trans-Golgi marker TGN46 as early as 20 minutes post-warming, and again at 40 minutes post-warming (Figs 8B, 9B, 10B and 10D). Trafficking of incoming bunyaviruses to the Golgi complex has not been previously reported. Interestingly, nascent bunyaviral proteins are assembled in the Golgi to form progeny virions [15], and our observation potentially revels a link between virus entry and the establishment of replication. Other viruses have been reported to require trafficking to the Golgi for entry, for example human papilloma virus 16 interacts with retromer to escape the early endosome and traffic to the *trans*-Golgi [52]. Further investigations will be needed to discern whether SFTSV virions require trafficking to the Golgi complex and if this has been an overlooked aspect of entry with other bunyaviruses.

Using the previous observations as a foundation, we sought to determine if pharmacological inhibition of UGCG altered the trafficking of incoming rVSV-SFTSV particles. At 20 minutes post-warming in NB-DNJ-treated and control cells, virions were observed to similarly populate EEA1+ endosomes and to a lesser extent, TGN46+ compartments. After 40 minutes post-warming, virus particles again co-stained with EEA1 and TGN46, but large punctate viral complexes were observed in NB-DNJ and NB-DGJ treated cells (Fig 9A and 9B, S7 Fig), while untreated cells contained only small punctate virion structures. Volumetric analysis of virus immunostaining in z-stack images revealed a statistically significant increase in the fluorescent volume of particle puncta in drug-treated cells compared to those in untreated cells, suggesting the accumulation of virions within a vesicular compartment (Fig 9C). The viral aggregates appeared to share similar markers to virions in untreated cells (EEA1, TGN46), suggesting that the virions are not overtly mislocalized. However, the composition of the compartment may be altered, thus preventing viral fusion and escape or further trafficking to a downstream location.

Investigations into the role of GlcCer on endocytic trafficking have found that depletion of GlcCer pools with PDMP or NB-DGJ treatment results in disrupted GSL recycling to the Golgi and leads to the accumulation of GSLs and cholesterol in lysosomes [53]. Furthermore, UGCG inhibition leads to increased lysosomal pH by up to 1 pH unit [54], potentially inhibiting the activity of pH-dependent lysosomal proteases and leading to the accumulation of cholesterol. The altered lipid composition of the lysosome due to GSL and cholesterol accumulation, increase in lysosomal pH, and disrupted recycling to the Golgi could, in a non-mutually exclusive manner, be causing the observed accumulation of rVSV-SFTSV virions in GlcCer depleted cells. Bunyavirus glycoproteins have been shown to undergo pH dependent conformational changes [55], and low pH as well as an unidentified serine protease are required for efficient SFTSV entry [20,21]. Future studies will investigate how changes to the endo-lysosomal network in GlcCer-depleted cells may be contributing to the observed accumulation of virions.

The *Phlebovirus* genus can be generally subdivided into the phlebotomine/mosquito-transmitted viruses, which includes Rift Valley fever virus, and the tick-transmitted viruses, for which the archetype is Uukuniemi virus. SFTSV forms a unique species within the tick-transmitted viruses, which also includes Heartland Virus (HRTV). While HRTV entry was sensitive to perturbations to UGCG, RVFV was unaffected. A recently published haploid genetic screen using the MP-12 strain of RVFV found glycosaminoglycan (GAG) biogenesis, and specifically heparan sulfate formation, as being important for RVFV infection [56]. The rVSV-SFTSV and RVFV haploid screens did not share any overlapping hits, which suggests that these two distantly related viruses have distinct entry requirements. This may be due to the difference in the arthropod vector. More extensive studies are underway to determine if the requirement for UGCG is limited to the SFTSV phlebovirus species, or extends to other tick-borne phleboviruses. Interestingly, ticks also encode a homolog of UGCG and are able to synthesize glycosphingolipids. Certain tick cells lines, including those belonging to the *Ixodidae* family, are amenable to siRNA knockdown and future investigations will assess whether UGCG is important for infection with SFTSV within its arthropod vector.

Ours is the first reported forward genetic screen seeking to identify host requirements for SFTSV entry. Our screen identified a crucial role for GlcCer formation in the efficient entry of SFTSV, with GlcCer depletion resulting in the aggregation of virus particles during the entry process. Further investigations are needed to fully characterize the trafficking of SFTSV virions using labeled particles and live-cell microscopy and to identify the compartment in which SFTSV fusion occurs.

## Materials & methods

### Cells, viruses, and chemicals

A549 and Vero E6 cells purchased from ATCC and U-2 OS cells kindly provided by Sara Cherry (University of Pennsylvania) were cultured in DMEM supplemented with 10% FBS (Sigma). HAP1 cells used in the genetic screen were provided by Thijn Brummelkamp (Netherlands Cancer Institute) and were cultured in IMDM supplemented with 15% FBS, L-glutamine, and P/S. HAP1 UGCG KO cells were purchased from Horizon Discovery and cultured as above. All mammalian cell lines were grown at 37°C with 5% CO_2_ unless otherwise stated. Replication-competent rVSV-SFTSV was generated as described below. VSV and rVSV-SFTSV stocks were propagated in Vero E6 cells. SV40 was a kind gift from Dr. James Alwine (U. Pennsylvania) and Rift Valley fever virus (strain MP-12) was generously provided by Dr. Robert Doms (U. Pennsylvania). The isolate of SFTSV used in this study was a plaque-purified cell culture-adapted strain, designated Hubei 29pp (HB29pp), was supplied to the Glasgow laboratory via the CDC Arbovirus Diseases Branch, Division of Vector-Borne Infectious Diseases, Fort Collins, CO, courtesy of Amy Lambert. Stocks of recombinant viruses were grown in Vero E6 cells at 37°C by infecting at a low multiplicity of infection (MOI) and harvesting the culture medium at 7 days post-infection.

All siRNAs were purchased from Ambion and resuspended according to the manufacture’s instructions. Anti-VSV M mouse polyclonal sera was kindly provided by Dr. Robert Doms (U. Pennsylvania) and anti-UGCG mouse monoclonal antibody (1E5) was purchased from Abnova. Rabbit mAbs to EEA1 (C45B10), Rab5 (C8B1), golgin97 (D8P2K) and Rab7 (D95F2) were purchased from Cell Signaling Technologies, rabbit sera to TGN46 was purchased from Novus Bio, and rabbit sera to LAMP-1 was purchased from Abcam. The UGCG inhibitor D,L-*threo*-PDMP (Cayman Chemicals) was dissolved in Argon-purged DMSO, aliquoted, and stored at -20°C under inert gas. Both N-butyldeoxynojirimycin-HCl and n-(N-butyl)deoxygalactonojirimycin (Toronto Research Chemicals) were dissolved in water, aliquoted, and stored at -20°C. All primers used for qPCR analysis are listed in the Primer Table (S2 Table).

### Generation of recombinant viruses and pseudotypes

Replication recombinant vesicular stomatitis viruses were cloned and infectious virus rescued as described previously with slight modifications [57]. Briefly, a codon-optimized version of the SFTSV M segment (strain HB29) was synthesized (ThermoFisher GeneArt) and cloned into a DNA plasmid of the VSV anti-genome between the M and L proteins, in place of VSV G. 293T cells were first infected with MVA expressing T7 polymerase for 1 hour, washed 1X, and then transfected with the T7-driven rVSV-SFTSV anti-genome plasmid and helper plasmids encoding VSV N, P, and L (all pCAGGS). Media was changed the following day, and 48 hours post-transfection supernatants were collected, transferred to Vero cells, and passaged 3 times before collection of virus stocks.

VSV-HRTV pseudotypes were generated as described previously [58]. A codon-optimized version of the HRTV M segment (strain MO-4) was synthesized (ThermoFisher GeneArt) and cloned into pCAGGS. 293T cells were transfected with pCAGGS-HRTV and 18 hours later transduced with VSV-ΔG-RFP pseudotypes previously complemented with VSV G. Since the pseudovirion genome does not encode a glycoprotein, progeny virions are complemented with HRTV G_N_-G_C_. Supernatants were collected 36 hours post-transduction, aliquoted and stored at -80°C.

### HAP1 genetic screen & statistical analysis

HAP1 cells were passaged, enriched for haploid cells, and mutagenized using a lentiviral gene-trap vector as described previously [27]. 75 millions cells from the HAP1 gene-trapped library were thawed, allowed to recover for 48 hours, then infected with rVSV-SFTSV (MOI 1) by spinfection (1200xg, 30m, 21°C). Surviving cells were allowed to expand for 14 days before being trypsinized and pooled. A portion of the cells were stored at -80°C for cellular assays, while DNA was extracted from the remaining cells for integration site analysis. Deep sequencing of lentiviral integration sites was conducted as described [30] with the exception that sonication rather than restriction enzymes was used to fragment genomic DNA and sequencing was performed using an Illumina MiSeq. The frequency of independent integration sites within each RefSeq annotated gene was determined for both the rVSV-SFTSV-selected population (N = 4,502) and the unselected library (N = 803,369). A Chi-Square Exact Test with False-Discover Rate correction was used to determine whether a given gene contained more integration sites in the selected population than expected based on the unselected library. A p-value of 0.05 or less was used as the cut-off for significance.

### RNAi infection experiments

For siRNA experiments, 10pmol of siRNA (Ambion) was co-incubated with 1.5μL of Lipofectamine RNAiMAX in 200μL of Opti-MEM for 20 minutes before the addition of 200,000 U-2 OS cells diluted in DMEM (10% FBS). The cell-transfection mixture was then plated into one well of a 12-well plate, re-plated the following day, and infected 72 hours post-transfection. Virus was diluted in DMEM (10% FBS) and used to spinfect cells (1200xg, 30 minutes, RT). Virus was added to achieve 15–30% infection of cells in negative control or untreated samples. Following a single-cycle infection (10 hours), cells were collected, fixed in 2% paraformaldehyde, immunostained for viral antigen, and the percentage of infected cells was quantified by flow cytometric analysis (≥10^4^ events).

Plasmids containing lentiviral shRNAs to UGCG or a Scrambled control were kindly provided by Dr. Daniel DiMaio (Yale University). Lentiviral pseudotypes were generated in 293T cells by co-transfection of pSIREN-shRNA, pSPAX, and pCAGGS-VSV G. Forty-eight hours post-transfection supernatants were collected and used to transduced U-2 OS cells. Following 4 days of puromycin selection, cells harboring the shRNA constructs were used for virus infection experiments as described above. In parallel with the harvesting of virus-infected cells, RNA extracts were collected according to manufacturer’s instructions (Qiagen RNeasy) to assess relative mRNA expression of genes of interest by RT-qPCR. Following cDNA synthesis (Invitrogen First-Strand Synthesis Kit) using random hexamers, qPCR reactions were set up using 10μL Power SYBR Green PCR Mastermix, 2μL cDNA, 2μL each Forward and Reverse primers (5μM), and 4μL water and run on a 7900HT Fast Real-Time PCR Machine (Applied Biosystems)

### UGCG inhibitor infections

A549 or U-2 OS cells were plated at 40,000 cells/well in 24-well dishes the evening prior to drug addition. The next morning, inhibitors were diluted in DMEM (10% FBS) and added to cells. Following pre-treatment for 24–72 hours (specific interval indicated in Figure Legend), virus was diluted in media containing drug and used to spinfect cells (1200g, 30min, RT). Virus was added to achieve 15–30% infection of cells in untreated samples. Virus and drug was left on cells during the course of infection. After a single replication cycle, cells were collected, fixed with paraformaldehyde, immunostained for viral antigen, and the percent of infected cells was quantified using flow cytometric analysis (≥10^4^ events).

### Virus titration by plaque assay

Vero E6 cells were infected with serial dilutions of virus and incubated under an overlay consisting of DMEM supplemented with 2% FCS and 0.6% Avicel (FMC BioPolymer) at 37°C for 5 days. Cell monolayers were fixed with 4% formaldehyde. Following fixation, cell monolayers were stained with Giemsa to visualize plaques.

### Binding & internalization assay

U-2 OS cells were either transfected with siRNAs 72 hours prior to the assay or pre-treated with NB-DNJ for 48 hours prior. Cells were first chilled to 4°C on ice for 30 minutes to inhibit endocytosis before media was replaced with rVSV-SFTSV or SV40 diluted in cold DMEM (2% FBS). Virus was allowed to bind to cells for 1 hour at 4°C, then cells either remained at 4°C for an additional hour or were moved to 37°C for the second hour to permit internalization. At the end of the second hour, cells kept at 4°C were either washed 3X with PBS and directly lysed, or washed, trypsinized for 10 minutes, washed 3X, and then lysed to measure levels of bound virus and background virus signal, respectively. Cells that were moved to 37°C were washed 3X, trypsinized to remove surface-bound virions, washed 3X, then lysed in order to determine levels of internalized virus. RNA (rVSV-SFTSV) or DNA (SV40) extractions were performed on cell lysates according to the manufacturer’s instructions (Qiagen). RNA from rVSV-SFTSV samples was reverse-transcribed with random hexamers using the First-Strand Synthesis Kit (Invitrogen) while DNA from SV40 samples was used directly for qPCR. qPCR reactions were run on a 7900HT Fast Real-Time PCR Machine (Applied Biosystems). Viral genomes were normalized to GAPDH by the 2^-ΔΔCt^ method [59].

### Immunofluorescence microscopy

A549 cells were plated on glass coverslips (10,000/well) the evening prior to initiating drug treatment. The following morning, N-butyldeoxynojirimycin-HCl (200μM) was added to cells for 48 hours prior to virus addition. In the case of siRNA transfection, U-2 OS cells were transfected as described as above and media was replaced 24 hours later. Cells were plated onto glass coverslips 48 hours post-transfection and virus added 72 hours post-transfection. Prior to virus addition, cells were chilled on ice at 4°C for 30 minutes to inhibit endocytosis. Media was then replaced with rVSV-SFTSV (MOI 50) diluted in cold DMEM (2% FBS) and bound to cells by centrifugation (1200xg, 30 minutes, 4°C). Following centrifugation, cells washed 1X with cold PBS, replaced with warm media (37°C), and placed in a 37°C incubator for various lengths of time (10–40 minutes). At indicated time points, cells were washed 3X with cold PBS then fixed with 2% paraformaldehyde for 10 minutes. Cells were washed 3X with PBS, permeabilized with 0.1% saponin for 10 minutes, then blocked for 30 minutes in PBST (0.1% Tween) with 1% BSA and 0.3M glycine. Coverslips were then incubated in primary antibody diluted in PBST and 1% BSA overnight at 4°C, then incubated with secondary antibody for 1 hour at room temperature before mounting onto glass slides. Cells were imaged at the CDB Microscopy Core (University of Pennsylvania) using a Leica DM6000 widefield microscope and Photometrics HQ2 CCD camera. Images were deconvolved using AutoQuant deconvolution package (Leica) and brightness/contrast was adjusted using FIJI (ImageJ). Volumetric analysis of fluorescent VSV-M stained puncta was performed using Volocity (Perkin Elmer). Intensity and size thresholds were set based on uninfected cells stained for viral antigen and excluded >99% of background events.

## Supporting information

S1 Fig RT-qPCR analysis of UGCG expression in shRNA treated cells. UGCG mRNA levels in cells expressing shScrambled and shUGCG were determined by RT-qPCR, normalized to GAPDH mRNA levels, and expressed relative to the scrambled shRNA. Mean ± S.E.M. for 2 independent experiments.
(TIFF)

S2 Fig Cell viability in the presence of UGCG pharmacological inhibitors. A549 cells were plated into 24 well plates and the next day UGCG inhibitors were added at various concentrations in triplicate. Following incubation with NB-DGJ or NB-DNJ for 48 hours (A) or PDMP for 24 hours (B), cells were collected, stained with trypan blue, and counted using an automated cell counter. Cell counts were normalized to the untreated control. Mean ± S.E.M. for two independent experiments.
(TIFF)

S3 Fig Cholera toxin staining of siRNA-treated cells to measure relative GM1 surface levels. Cholera toxin subunit B (CtxB) binds to cellular GM1 to mediate uptake and can be used to measure GM1 surface levels. U2OS cells were transfected with siRNAs targeting UGCG, LCS, ganglioside biosynthetic enzymes, or a non-targeting control. 72 hours post-transfection, cells were collected, chilled on ice for 10 minutes, then incubated with fluorescently tagged CtxB for 15 minutes on ice, washed 3X, and CtxB binding was analyzed using a flow cytometer. Values are expressed relative to the negative control siRNA.
(TIFF)

S4 Fig Alignment of SFTSV and Heartland virus glycoprotein precursors. Amino acid alignment of SFTSV (strain HB29) and HRTV (strain MO-4) glycoprotein polyprotein precursors. ClustalW alignment with BLOSUM cost matrix was performed within Geneious software package (Biomatters Ltd).
(TIFF)

S5 Fig Immunofluorescence microscopy of incoming rVSV-SFTSV virions with markers for Rab7 and LAMP1, 20 minutes. (**A, B**) A549 cells were plated onto glass coverslips and the following day replaced with media containing NB-DNJ (200μM) or left untreated. Forty-eight hours later, cells were chilled to 4°C on ice for 30 minutes, then rVSV-SFTSV was diluted in 250μL of cold media and bound to the surface of cells by centrifugation (1200xg, 30’, 4°C). Following centrifugation, media was replaced with pre-warmed media (37°C) and the cells placed in a 37°C incubator for 20 minutes before fixation in 2% paraformaldehyde for 10 minutes. Cells were then immunostained for viral antigen (anti-VSV M, red), Rab7 (A) or LAMP1 (B) (green), and nuclei stained with DAPI (blue). Coverslips were then mounted on glass slides and imaged using a widefield microscope (Leica D6000) with deconvolution. Images are representative from at least 3 independent experiments. Scale bar represents 5μm.
(TIFF)

S6 Fig Immunofluorescence microscopy of incoming rVSV-SFTSV virions with markers for Rab7 and LAMP1, 40 minutes. (**A, B**) A549 cells were plated onto glass coverslips and the following day replaced with media containing NB-DNJ (200μM) or left untreated. Forty-eight hours later, cells were chilled to 4°C on ice for 30 minutes, then rVSV-SFTSV was diluted in 250μL of cold media and bound to the surface of cells by centrifugation (1200xg, 30’, 4°C). Following centrifugation, media was replaced with pre-warmed media (37°C) and the cells placed in a 37°C incubator for 40 minutes before fixation in 2% paraformaldehyde for 10 minutes. Cells were then immunostained for viral antigen (anti-VSV M, red), Rab7 (A) or LAMP1 (B) (green), and nuclei stained with DAPI (blue). Coverslips were then mounted on glass slides and imaged using a widefield microscope (Leica D6000) with deconvolution. Images are representative from at least 3 independent experiments. Scale bar represents 5μm.
(TIFF)

S7 Fig Inhibition of UGCG using imino surge derivatives NB-DNJ and NB-DGJ leads to accumulation of rVSV-SFTSV virions at late time points. A549 cells were plated on glass coverslips and the following day n-butyldeoxynojirimycin (NB-DNJ) or N-(n-Butyl)deoxygalactonojirimycin (NB-DGJ) (200μM) was added. Forty-eight hours later, cells were chilled to 4°C on ice for 30 minutes, then rVSV-SFTSV was diluted in 250μL of cold media and bound to the surface of cells by centrifugation (1200xg, 30’, 4°C). Following centrifugation, media was replaced with pre-warmed media (37°C) and the cells placed in a 37°C incubator for 40 minutes before fixation in 2% paraformaldehyde for 10 minutes. Cells were then immunostained for viral antigen (anti-VSV M, red) and nuclei were stained with DAPI (blue). Coverslips were then mounted on glass slides and imaged using a widefield microscope (Leica D600) with deconvolution. Images are representative from at least 2 independent experiments. Scale bar represents 5μm.
(TIFF)

S8 Fig NB-DNJ treatment leads to accumulation of rVSV-SFTSV virions but not VSV virions at late time points. (**A, B**) A549 cells were plated onto glass coverslips and the following day replaced with media containing NB-DNJ (200μM) or left untreated. Forty-eight hours later, cells were chilled to 4°C on ice for 30 minutes, then rVSV-SFTSV (A) or VSV (B) was diluted in 250μL of cold media and bound to the surface of cells by centrifugation (1200xg, 30’, 4°C). Following centrifugation, media was replaced with pre-warmed media (37°C) and the cells placed in a 37°C incubator for 40 minutes before fixation in 2% paraformaldehyde for 10 minutes. Cells were then immunostained for viral antigen (anti-VSV M, red) and nuclei stained with DAPI (blue). Coverslips were then mounted on glass slides and imaged using a widefield microscope (Leica D6000) with deconvolution. Images are representative from at least 3 independent experiments. Scale bar represents 5μm.
(TIFF)

S9 Fig Immunofluorescence microscopy of rVSV-SFTSV particles with Rab5 and golgin97 co-staining following UGCG knockdown. **(A-D)** U-2 OS cells were transfected with siRNAs targeting UGCG (siUGCG) or a non-targeting control (siNegCtrl) and plated onto glass coverslips. At 72 hours post-transfection cells were chilled to 4°C on ice and rVSV-SFTSV was bound by centrifugation (1200xg, 30’, 4°C). Following centrifugation, media was replaced with pre-warmed media (37°C) and the cells placed in a 37°C incubator for 20 or 40 minutes before fixation in 1% paraformaldehyde for 15 minutes. Cells were then immunostained for viral antigen (anti-VSV M, red), cellular markers (green), and nuclei stained with DAPI (blue). Images are representative from at least 3 independent experiments. (**A,B**) U-2 OS cells fixed after 20 minutes were co-stained for rVSV-SFTSV (red) and Rab5 (A) or golgin97 (B) (green). (**C,D**) U-2 OS cells fixed after 40 minutes and stained as above. Scale bar represents 5μm.
(TIFF)

S10 Fig Immunofluorescence microscopy of incoming rVSV-SFTSV virions with Rab7 co-staining, 20 and 40 minutes. **(A,B)** U-2 OS cells were transfected with siRNAs targeting UGCG (siUGCG) or a negative control (siNeg Ctrl) and 48 hours later plated onto glass coverslips. At 72 hours post-transfection, cells were chilled to 4°C for 30 minutes before rVSV-SFTSV (MOI 50) diluted to 250μL in cold media was added and bound to cells by centrifugation (1200xg, 30’, 4°C). Following centrifugation, media was replaced with pre-warmed media (37°C) and the cells placed in a 37°C incubator for 20 minutes (A) or 40 minutes (B) before fixation in 1% paraformaldehyde for 15 minutes. Cells were then immunostained for viral antigen (anti-VSV M, red) and Rab7 (green), while nuclei were stained with DAPI (blue). Coverslips were then mounted on glass slides and imaged using a widefield microscope (Leica D6000) with deconvolution. Images are representative of at least 3 independent experiments. Scale bar represents 5μm.
(TIFF)

S11 Fig Ganglioside pre-treatment does not neutralize infection rVSV-SFTSV. Virus was diluted to equal volume in DMEM (1% FBS) then mixed gangliosides (from bovine brain, Sigma) were added in the amounts indicated and incubated for 1 hour at room temperature before being overlaid onto U2OS cells. Following a single cycle infection (rVSV-SFTSV and VSV–10hr, SV4–24hr), cells were harvested, fixed, immunostained for viral antigen, and percent infection was quantified using flow cytometry. Infection levels are expressed relative to the no ganglioside control. Mean ± S.D. for 2 independent experiments.
(TIFF)

S1 Table Genes identified in rVSV-SFTSV selected population from HAP1 screen. Gene symbol, number of independent gene-trap integrations within genes for selected and control populations, number of total integrations mapped in selected and control populations, false-discovery rate corrected p-value. Ranked by p-value.
(XLSX)

S2 Table Primers used for qPCR analysis. Forward and reverse primers used for qPCR analysis of gene expression and viral genomes. Probe sequence provided when necessary for analyses using TaqMan mastermix. SYBR green mastermix used for all other circumstances.
(TIFF)
