# Supplementary figures and images for: A role for glycolipid biosynthesis in severe fever with thrombocytopenia syndrome virus entry
Source: PLoS Pathog. 2017 Apr 7;13(4):e1006316. doi: 10.1371/journal.ppat.1006316 (PMC5397019; doi:10.1371/journal.ppat.1006316)

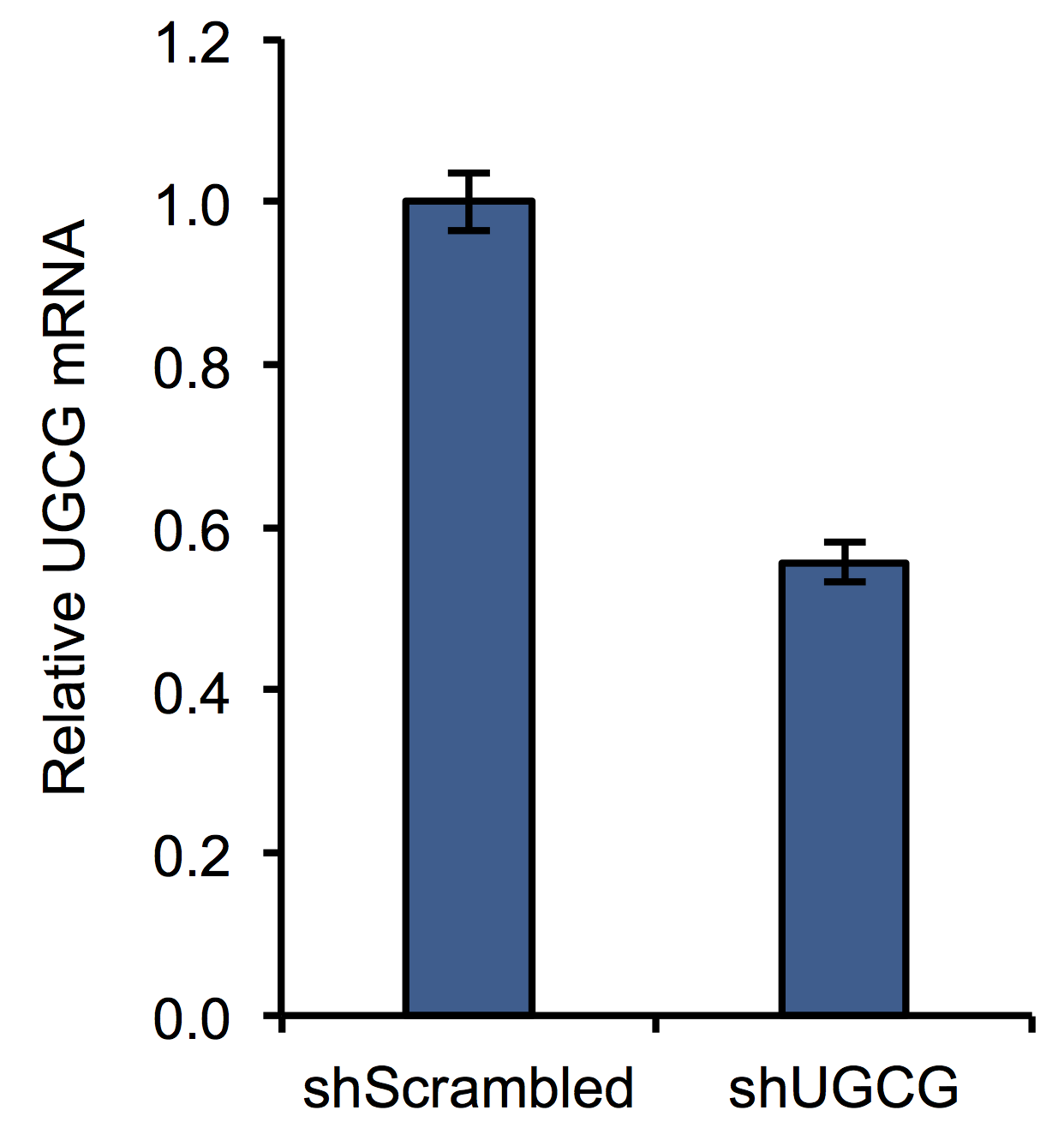

Supplement: S1 Fig — UGCG mRNA levels in cells expressing shScrambled and shUGCG were determined by RT-qPCR, normalized to GAPDH mRNA levels, and expressed relative to the scrambled shRNA. Mean ± S.E.M. for 2 independent experiments. (TIFF) [file ppat.1006316.s001.tiff]

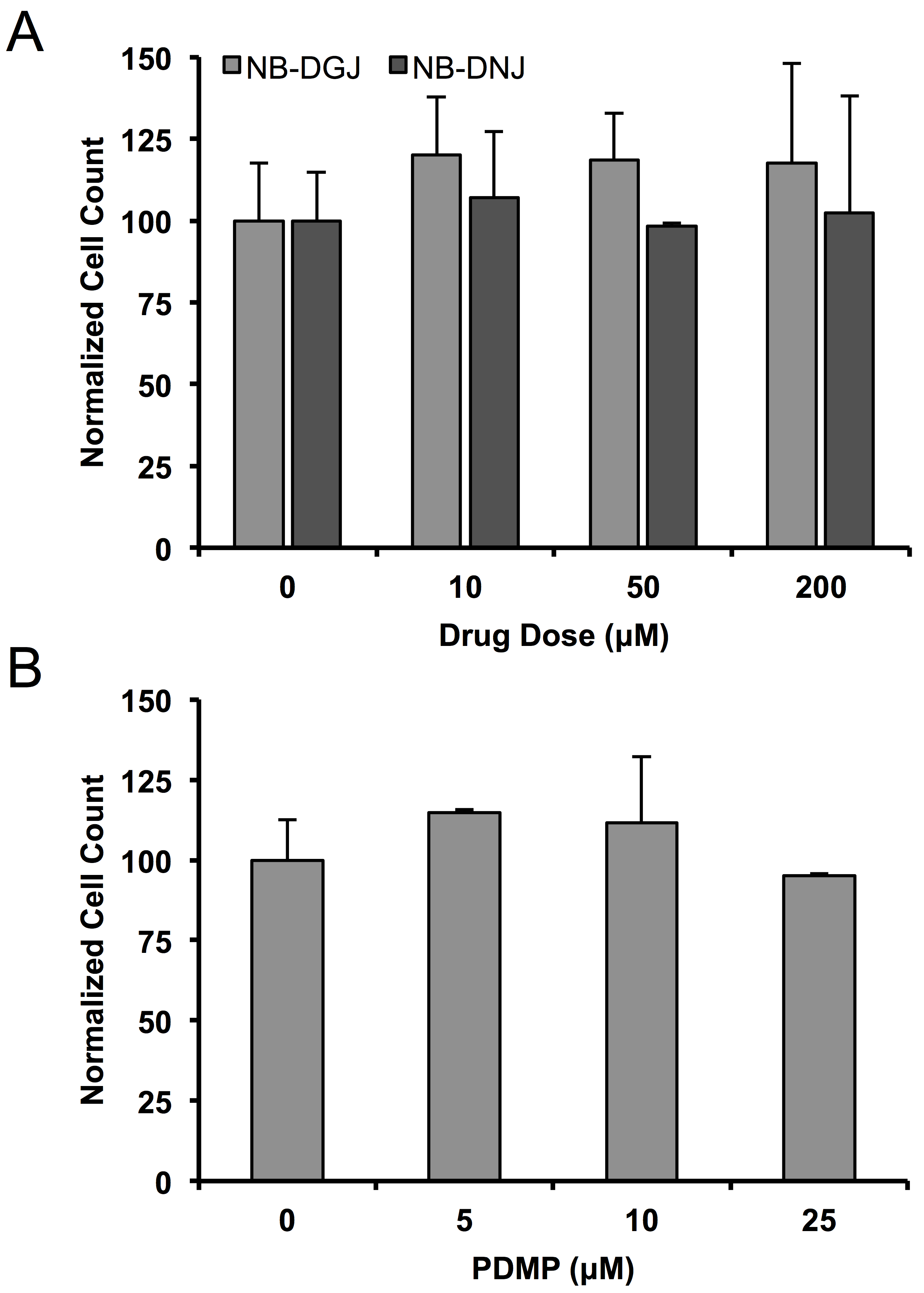

Supplement: S2 Fig — A549 cells were plated into 24 well plates and the next day UGCG inhibitors were added at various concentrations in triplicate. Following incubation with NB-DGJ or NB-DNJ for 48 hours (A) or PDMP for 24 hours (B), cells were collected, stained with trypan blue, and counted using an automated cell counter. Cell counts were normalized to the untreated control. Mean ± S.E.M. for two independent experiments. (TIFF) [file ppat.1006316.s002.tiff]

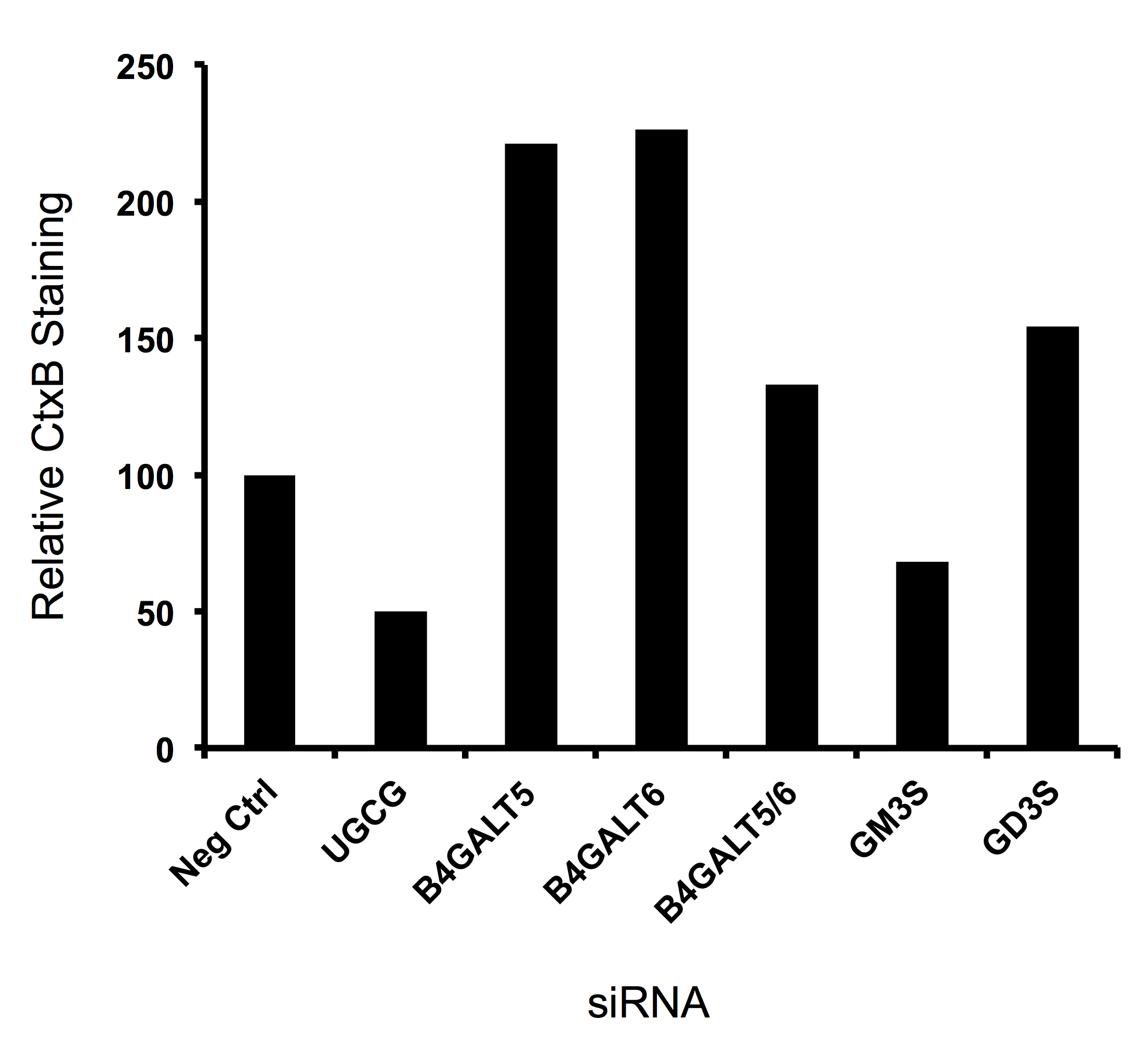

Supplement: S3 Fig — Cholera toxin subunit B (CtxB) binds to cellular GM1 to mediate uptake and can be used to measure GM1 surface levels. U2OS cells were transfected with siRNAs targeting UGCG, LCS, ganglioside biosynthetic enzymes, or a non-targeting control. 72 hours post-transfection, cells were collected, chilled on ice for 10 minutes, then incubated with fluorescently tagged CtxB for 15 minutes on ice, washed 3X, and CtxB binding was analyzed using a flow cytometer. Values are expressed relative to the negative control siRNA. (TIFF) [file ppat.1006316.s003.tiff]

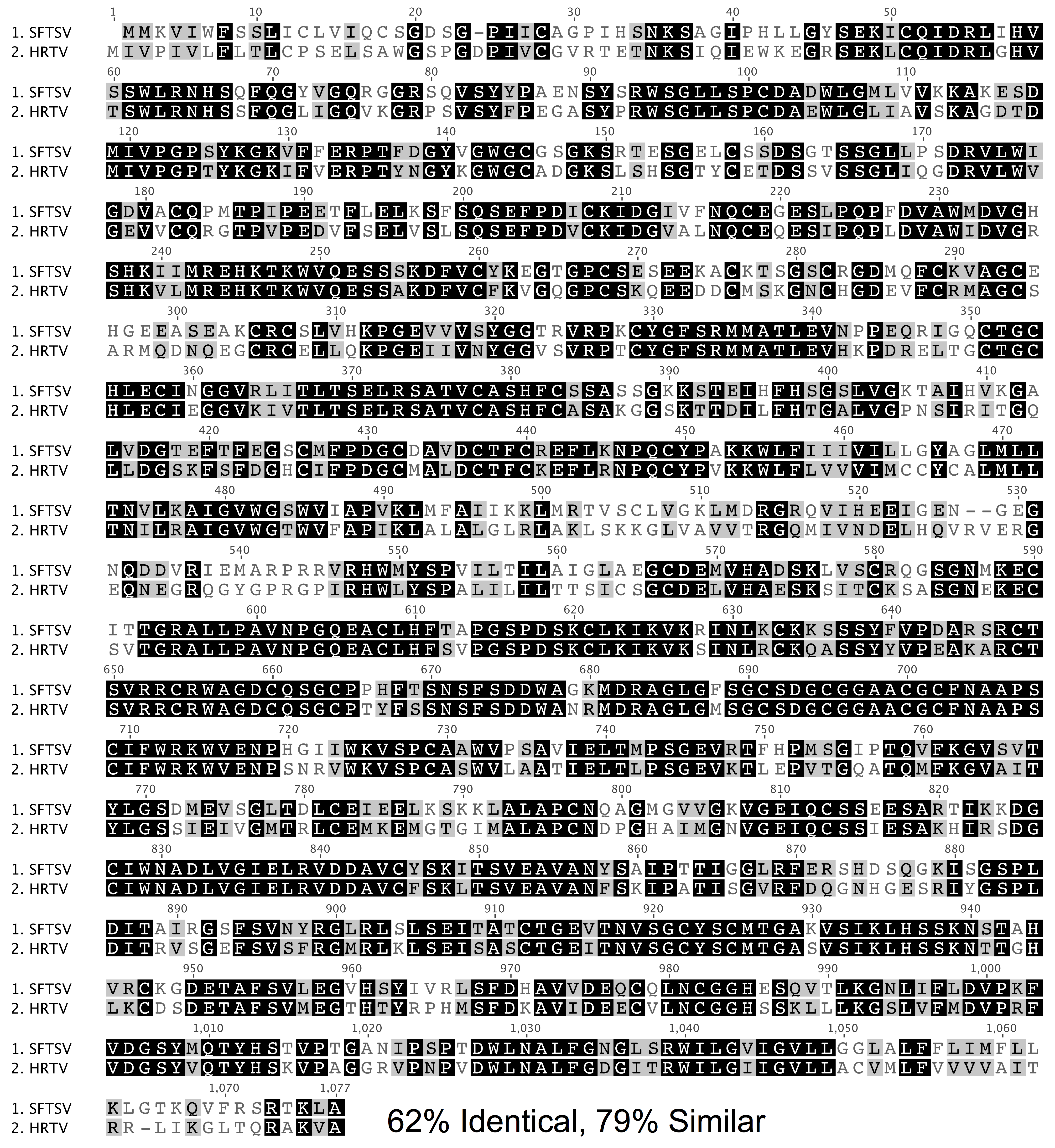

Supplement: S4 Fig — Amino acid alignment of SFTSV (strain HB29) and HRTV (strain MO-4) glycoprotein polyprotein precursors. ClustalW alignment with BLOSUM cost matrix was performed within Geneious software package (Biomatters Ltd). (TIFF) [file ppat.1006316.s004.tiff]

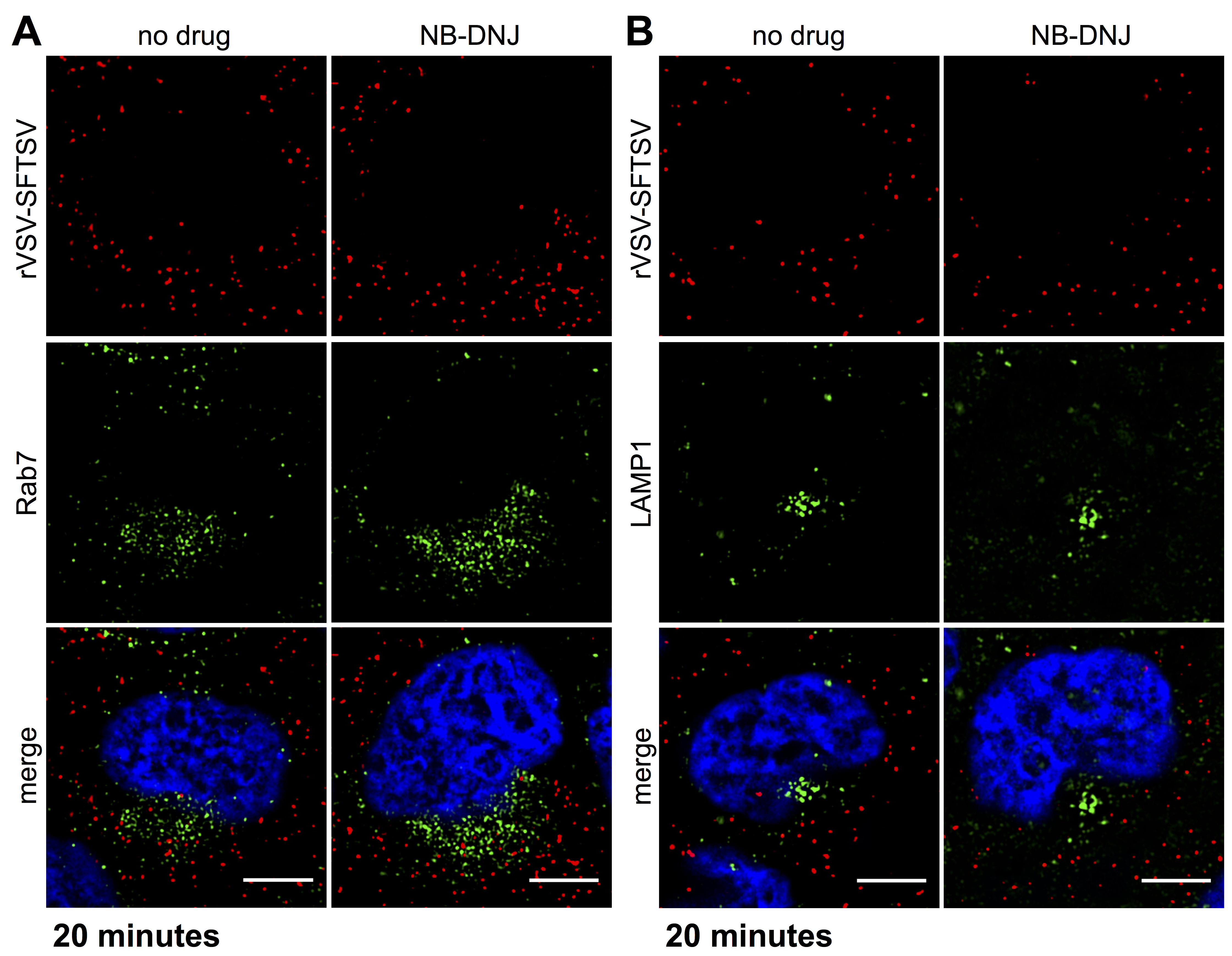

Supplement: S5 Fig — (A, B) A549 cells were plated onto glass coverslips and the following day replaced with media containing NB-DNJ (200μM) or left untreated. Forty-eight hours later, cells were chilled to 4°C on ice for 30 minutes, then rVSV-SFTSV was diluted in 250μL of cold media and bound to the surface of cells by centrifugation (1200xg, 30’, 4°C). Following centrifugation, media was replaced with pre-warmed media (37°C) and the cells placed in a 37°C incubator for 20 minutes before fixation in 2% paraformaldehyde for 10 minutes. Cells were then immunostained for viral antigen (anti-VSV M, red), Rab7 (A) or LAMP1 (B) (green), and nuclei stained with DAPI (blue). Coverslips were then mounted on glass slides and imaged using a widefield microscope (Leica D6000) with deconvolution. Images are representative from at least 3 independent experiments. Scale bar represents 5μm. (TIFF) [file ppat.1006316.s005.tiff]

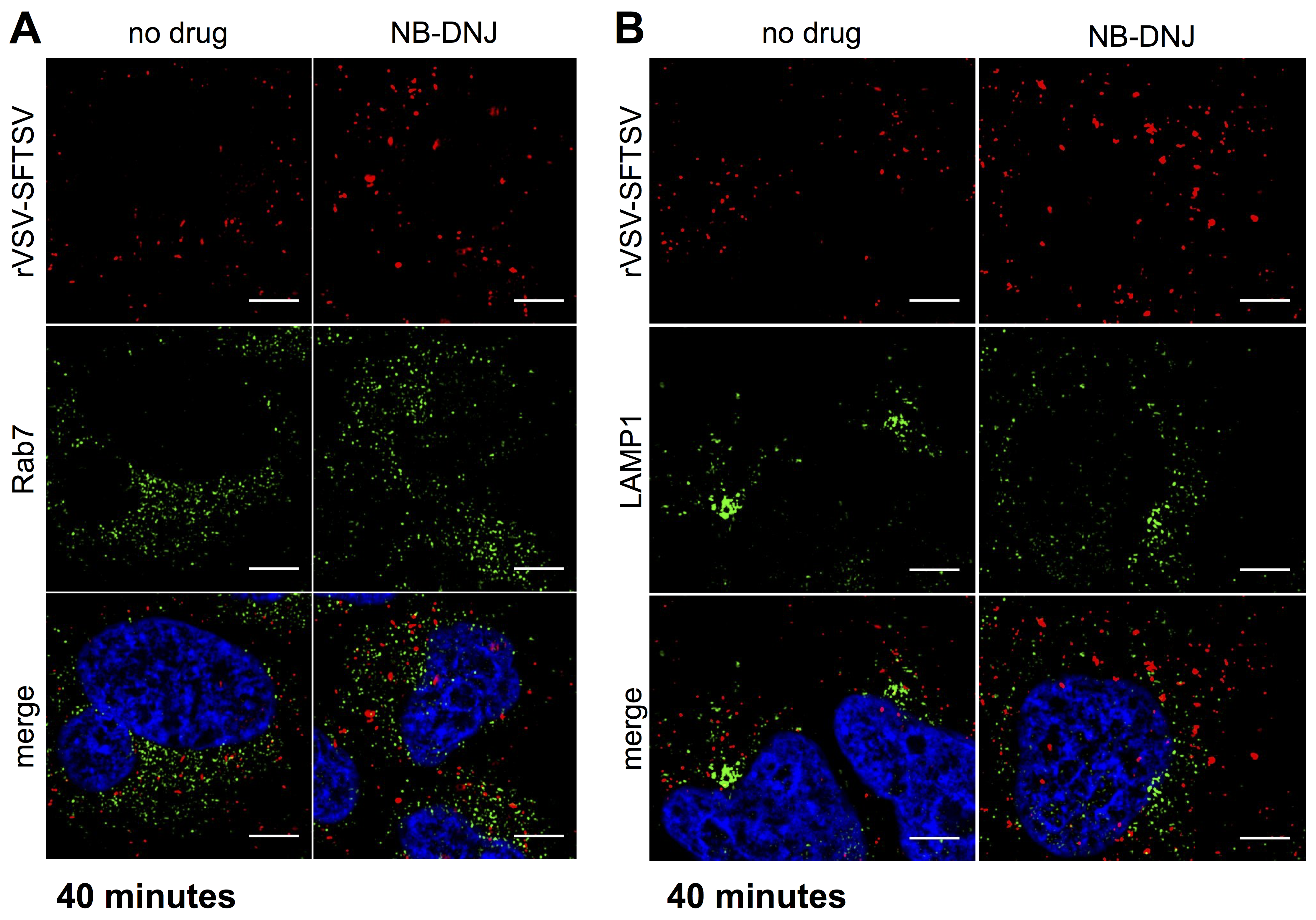

Supplement: S6 Fig — (A, B) A549 cells were plated onto glass coverslips and the following day replaced with media containing NB-DNJ (200μM) or left untreated. Forty-eight hours later, cells were chilled to 4°C on ice for 30 minutes, then rVSV-SFTSV was diluted in 250μL of cold media and bound to the surface of cells by centrifugation (1200xg, 30’, 4°C). Following centrifugation, media was replaced with pre-warmed media (37°C) and the cells placed in a 37°C incubator for 40 minutes before fixation in 2% paraformaldehyde for 10 minutes. Cells were then immunostained for viral antigen (anti-VSV M, red), Rab7 (A) or LAMP1 (B) (green), and nuclei stained with DAPI (blue). Coverslips were then mounted on glass slides and imaged using a widefield microscope (Leica D6000) with deconvolution. Images are representative from at least 3 independent experiments. Scale bar represents 5μm. (TIFF) [file ppat.1006316.s006.tiff]

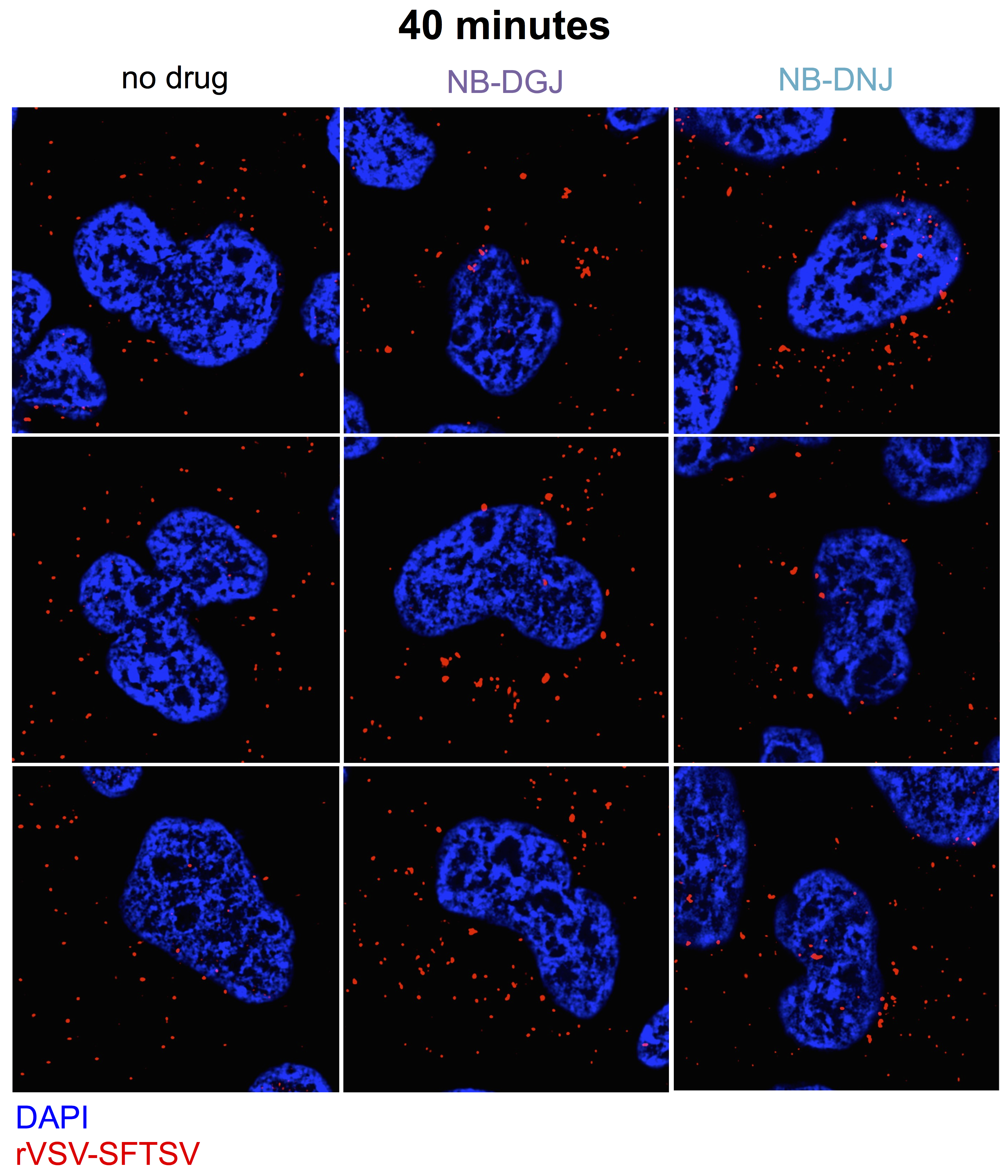

Supplement: S7 Fig — A549 cells were plated on glass coverslips and the following day n-butyldeoxynojirimycin (NB-DNJ) or N-(n-Butyl)deoxygalactonojirimycin (NB-DGJ) (200μM) was added. Forty-eight hours later, cells were chilled to 4°C on ice for 30 minutes, then rVSV-SFTSV was diluted in 250μL of cold media and bound to the surface of cells by centrifugation (1200xg, 30’, 4°C). Following centrifugation, media was replaced with pre-warmed media (37°C) and the cells placed in a 37°C incubator for 40 minutes before fixation in 2% paraformaldehyde for 10 minutes. Cells were then immunostained for viral antigen (anti-VSV M, red) and nuclei were stained with DAPI (blue). Coverslips were then mounted on glass slides and imaged using a widefield microscope (Leica D600) with deconvolution. Images are representative from at least 2 independent experiments. Scale bar represents 5μm. (TIFF) [file ppat.1006316.s007.tiff]

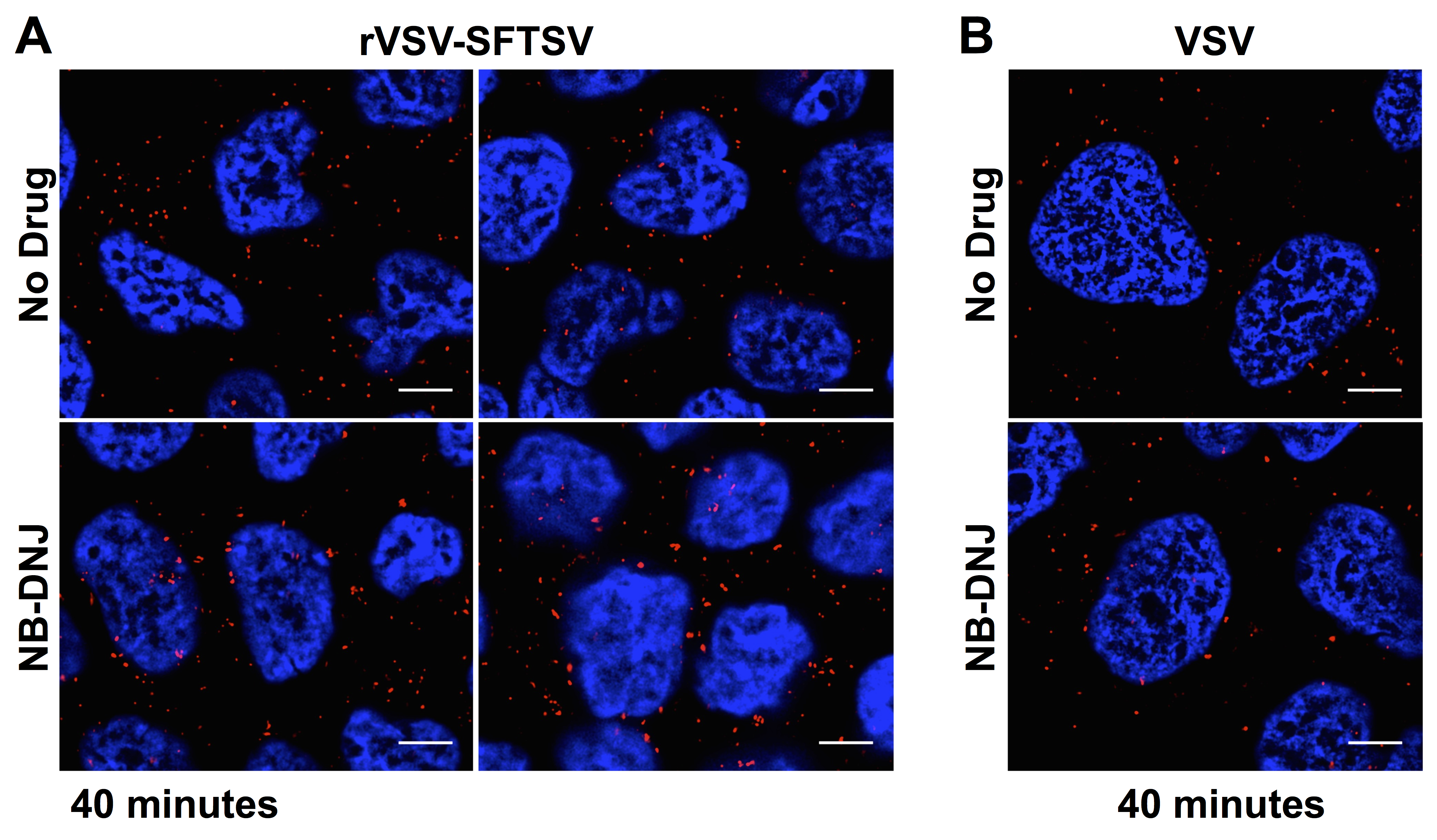

Supplement: S8 Fig — (A, B) A549 cells were plated onto glass coverslips and the following day replaced with media containing NB-DNJ (200μM) or left untreated. Forty-eight hours later, cells were chilled to 4°C on ice for 30 minutes, then rVSV-SFTSV (A) or VSV (B) was diluted in 250μL of cold media and bound to the surface of cells by centrifugation (1200xg, 30’, 4°C). Following centrifugation, media was replaced with pre-warmed media (37°C) and the cells placed in a 37°C incubator for 40 minutes before fixation in 2% paraformaldehyde for 10 minutes. Cells were then immunostained for viral antigen (anti-VSV M, red) and nuclei stained with DAPI (blue). Coverslips were then mounted on glass slides and imaged using a widefield microscope (Leica D6000) with deconvolution. Images are representative from at least 3 independent experiments. Scale bar represents 5μm. (TIFF) [file ppat.1006316.s008.tiff]

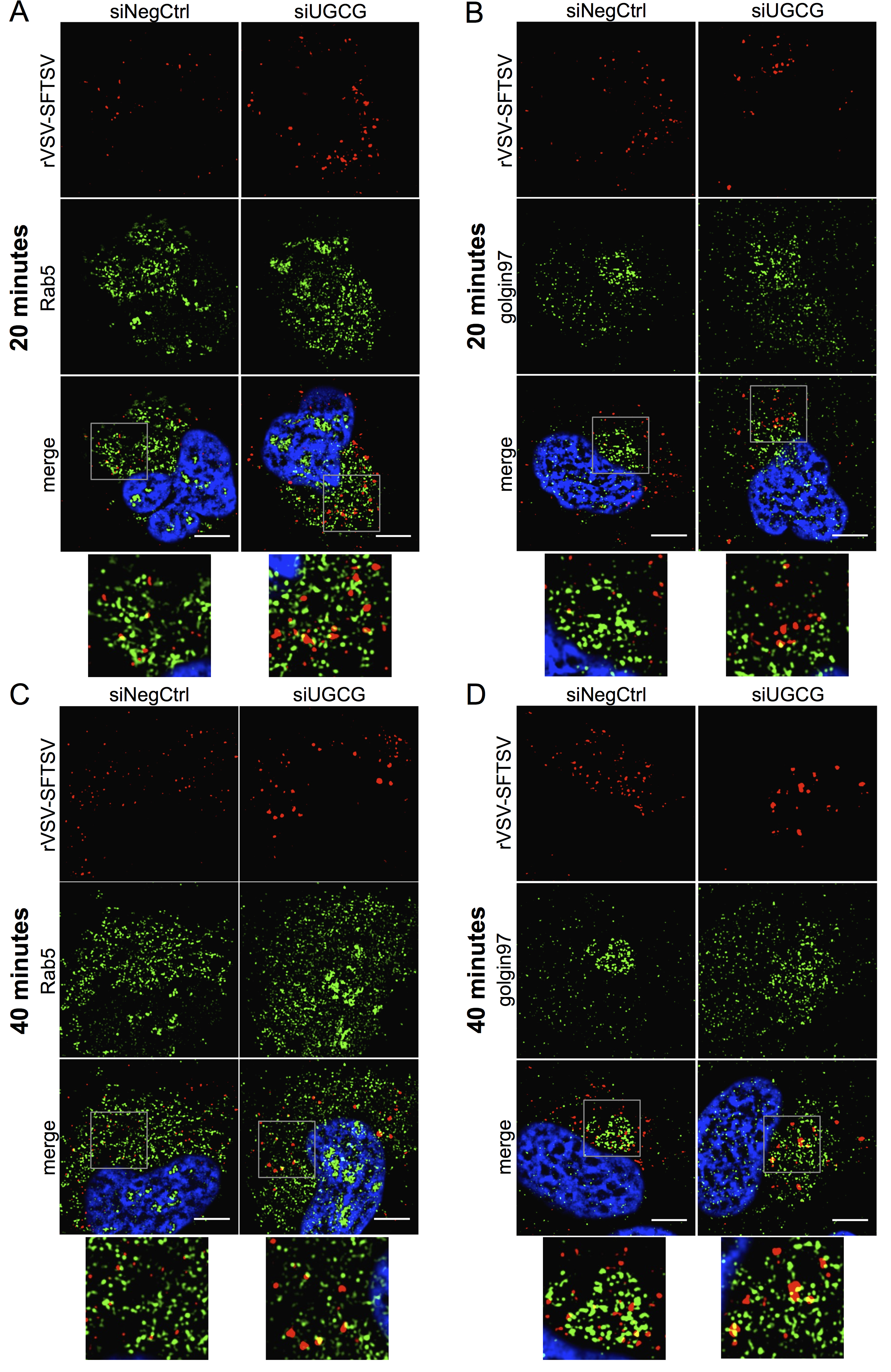

Supplement: S9 Fig — (A-D) U-2 OS cells were transfected with siRNAs targeting UGCG (siUGCG) or a non-targeting control (siNegCtrl) and plated onto glass coverslips. At 72 hours post-transfection cells were chilled to 4°C on ice and rVSV-SFTSV was bound by centrifugation (1200xg, 30’, 4°C). Following centrifugation, media was replaced with pre-warmed media (37°C) and the cells placed in a 37°C incubator for 20 or 40 minutes before fixation in 1% paraformaldehyde for 15 minutes. Cells were then immunostained for viral antigen (anti-VSV M, red), cellular markers (green), and nuclei stained with DAPI (blue). Images are representative from at least 3 independent experiments. (A,B) U-2 OS cells fixed after 20 minutes were co-stained for rVSV-SFTSV (red) and Rab5 (A) or golgin97 (B) (green). (C,D) U-2 OS cells fixed after 40 minutes and stained as above. Scale bar represents 5μm. (TIFF) [file ppat.1006316.s009.tiff]

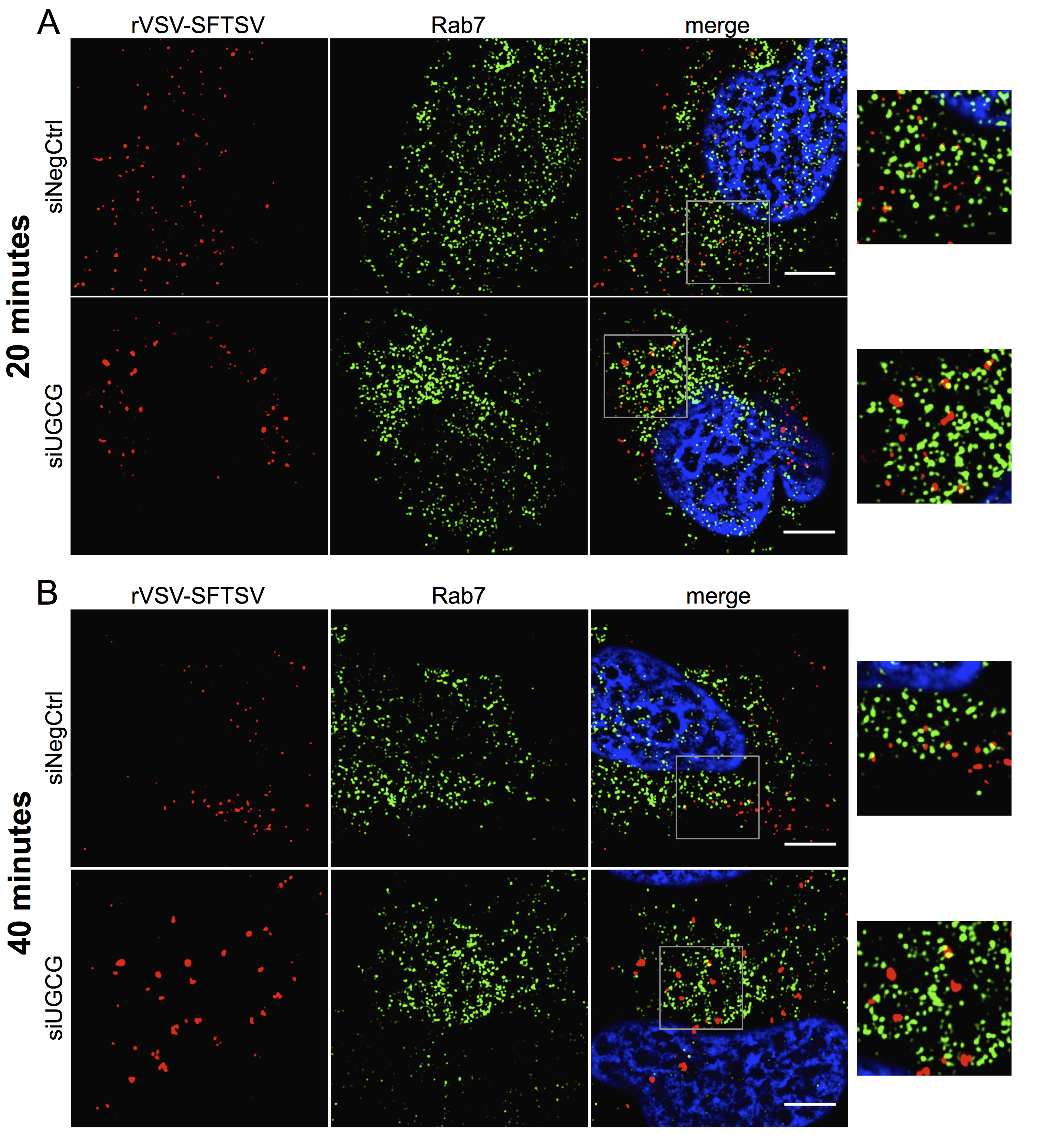

Supplement: S10 Fig — (A,B) U-2 OS cells were transfected with siRNAs targeting UGCG (siUGCG) or a negative control (siNeg Ctrl) and 48 hours later plated onto glass coverslips. At 72 hours post-transfection, cells were chilled to 4°C for 30 minutes before rVSV-SFTSV (MOI 50) diluted to 250μL in cold media was added and bound to cells by centrifugation (1200xg, 30’, 4°C). Following centrifugation, media was replaced with pre-warmed media (37°C) and the cells placed in a 37°C incubator for 20 minutes (A) or 40 minutes (B) before fixation in 1% paraformaldehyde for 15 minutes. Cells were then immunostained for viral antigen (anti-VSV M, red) and Rab7 (green), while nuclei were stained with DAPI (blue). Coverslips were then mounted on glass slides and imaged using a widefield microscope (Leica D6000) with deconvolution. Images are representative of at least 3 independent experiments. Scale bar represents 5μm. (TIFF) [file ppat.1006316.s010.tiff]

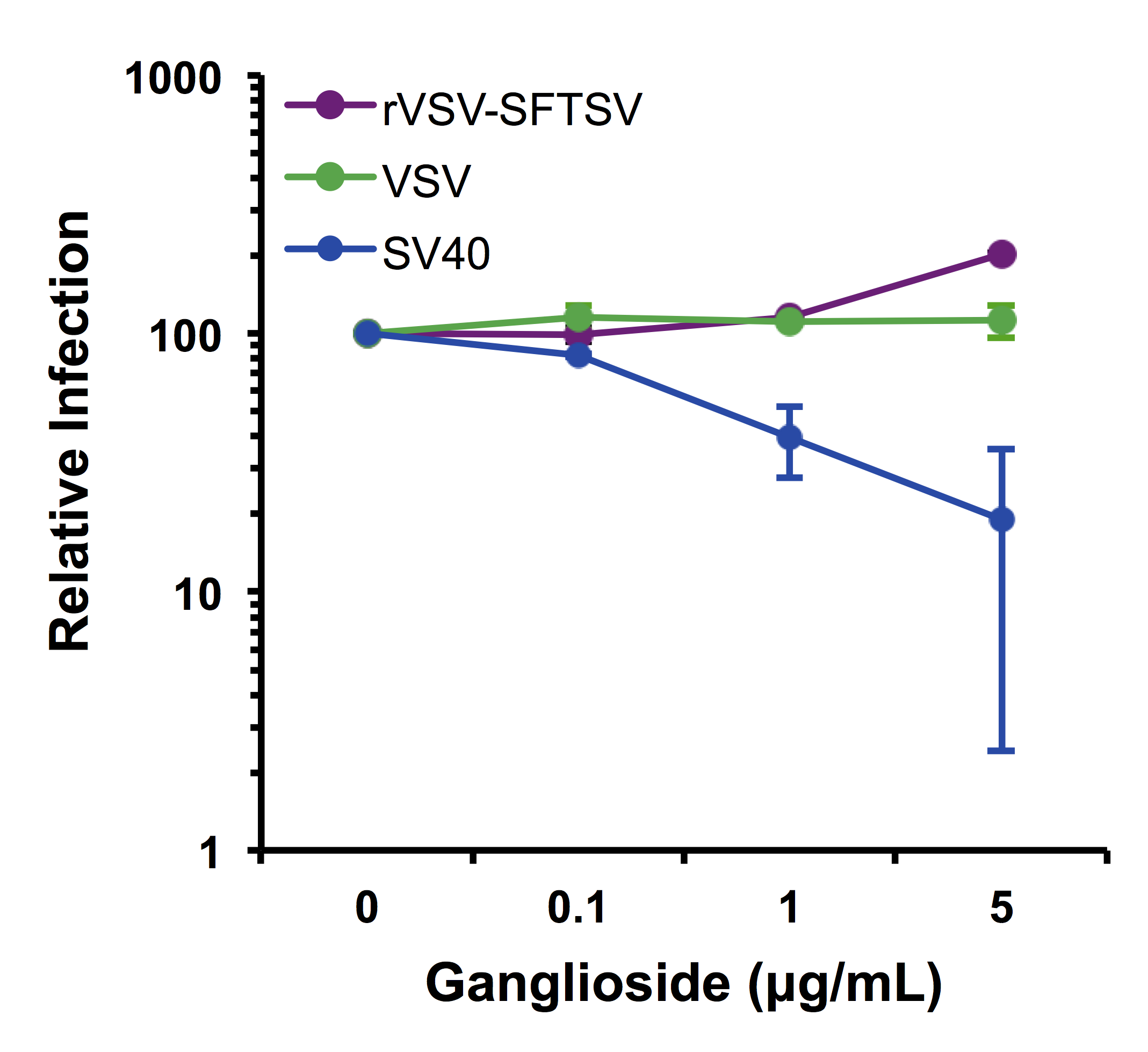

Supplement: S11 Fig — Virus was diluted to equal volume in DMEM (1% FBS) then mixed gangliosides (from bovine brain, Sigma) were added in the amounts indicated and incubated for 1 hour at room temperature before being overlaid onto U2OS cells. Following a single cycle infection (rVSV-SFTSV and VSV–10hr, SV4–24hr), cells were harvested, fixed, immunostained for viral antigen, and percent infection was quantified using flow cytometry. Infection levels are expressed relative to the no ganglioside control. Mean ± S.D. for 2 independent experiments. (TIFF) [file ppat.1006316.s011.tiff]

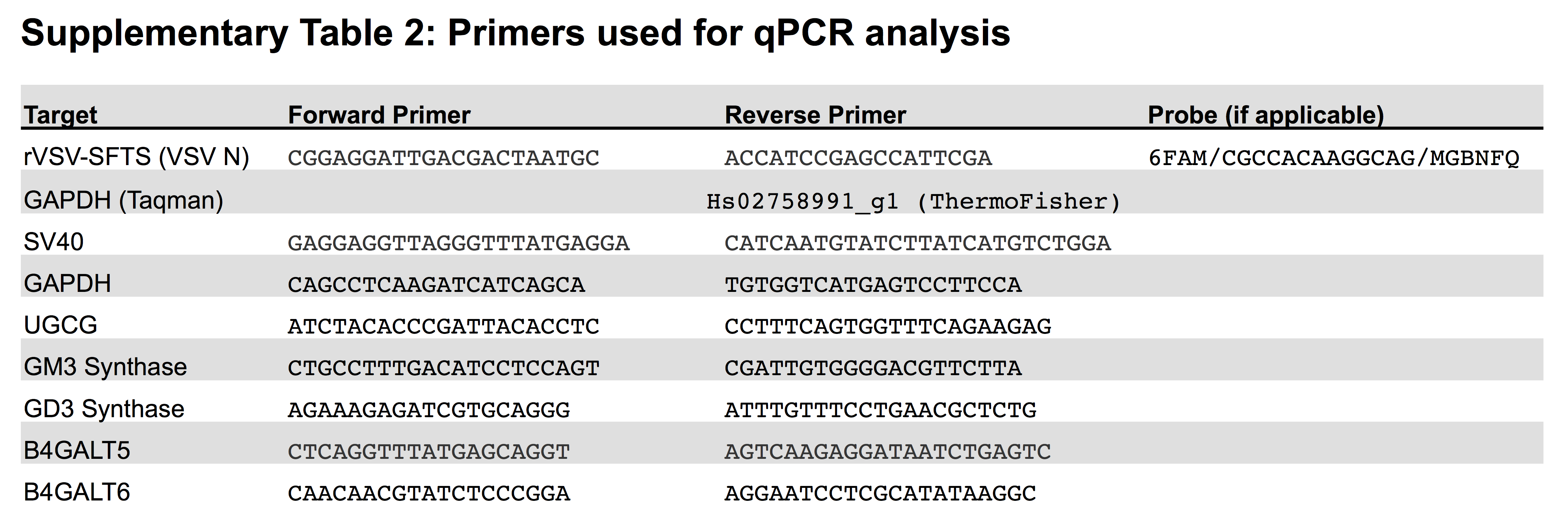

Supplement: S2 Table — Forward and reverse primers used for qPCR analysis of gene expression and viral genomes. Probe sequence provided when necessary for analyses using TaqMan mastermix. SYBR green mastermix used for all other circumstances. (TIFF) [file ppat.1006316.s013.tiff]
